# Ecological constraints increase the climatic debt in forests

**DOI:** 10.1038/ncomms12643

**Published:** 2016-08-26

**Authors:** Romain Bertrand, Gabriela Riofrío-Dillon, Jonathan Lenoir, Jacques Drapier, Patrice de Ruffray, Jean-Claude Gégout, Michel Loreau

**Affiliations:** 1 CNRS, Centre for Biodiversity Theory and Modelling, Theoretical and Experimental Ecology Station, UMR5321 CNRS-Université Paul Sabatier Toulouse III, 2 route du CNRS, FR-09200 Moulis, France; 2 LERFoB, INRA, AgroParisTech, FR-54000 Nancy, France; 3 Ecologie et Dynamique des Systèmes Anthropisés, FRE3498 CNRS-UPJV, 1 Rue des Louvels, FR-80037 Amiens Cedex 1, France; 4 IGN, Laboratoire de l Inventaire Forestier, 11 rue de l'Ile de Corse, FR-54000 Nancy, France; 5 CNRS, Institut de Biologie Moléculaire des Plantes, Université de Strasbourg,, 12 rue du Général Zimmer, FR-67084 Strasbourg Cedex, France

## Abstract

Biodiversity changes are lagging behind current climate warming. The underlying determinants of this climatic debt are unknown and yet critical to understand the impacts of climate change on the present biota and improve forecasts of biodiversity changes. Here we assess determinants of climatic debt accumulated in French forest herbaceous plant communities between 1987 and 2008 (that is, a 1.05 °C mean difference between the observed and bioindicated temperatures). We show that warmer baseline conditions predispose plant communities to larger climatic debts, and that climate warming exacerbates this response. Forest plant communities, however, are absorbing part of the temperature increase mainly through the species' ability to tolerate changing climate. As climate warming is expected to accelerate during the twenty-first century, plant migration and tolerance to climatic stresses probably will be insufficient to absorb this impact posing threats to the sustainability of forest plant communities.

## Abstract (web summary)

Many species show a time-lagged response to climate change, a phenomenon called climatic debt. Here, Bertrand and colleagues show that climate severity and plant tolerance to climate warming mainly influence the climatic debt of forest herbaceous plant communities.

## Graphical abstract

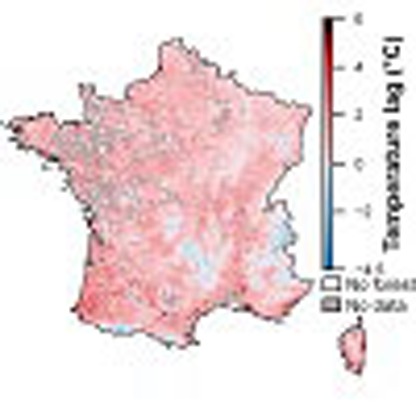

Current climate change has an impact on biodiversity from genes to the biosphere^1^, with widespread evidence of shifts in phenology, species distribution, community composition and ecosystem functioning and services^2^,^3^,^4^. However, recent studies^5^,^6^,^7^,^8^,^9^ have demonstrated that biotic responses observed in nature do not match those expected under the assumption of complete synchrony with climate change, leading to a disequilibrium^10^ or lag^11^ that generates a climatic debt^6^,^9^. In particular, the equilibrium that existed between temperature conditions and herbaceous plant assemblages in French forests has been disrupted since 1987 by the contemporary climate warming, leading to a larger climatic debt in lowland than in highland forests^8^. Time lags in biotic responses to historical climate changes have been documented (notably during interglacial periods), suggesting that climatic debts are common in nature and particularly so in forests^11^. The current climatic debt is emerging as a crucial ecological and conservation issue^6^,^8^,^9^,^10^,^12^ because of the unprecedented velocity of current climate change^13^,^14^, which is likely to lead to severe biodiversity loss in the twenty-first century.

The climatic debt is an integrative measure of the disequilibrium of communities with climate change^15^, but it is also affected by biotic, anthropogenic and other abiotic determinants^10^,^12^,^16^. Therefore, it offers an opportunity to move beyond the apparent impact of climate change to assess the multiple mechanisms underlying biodiversity changes^12^,^17^. Although a few recent studies explored some of the potential processes underlying the climatic debt in forest plant communities^18^,^19^,^20^, they focused on a handful of potential determinants, thus preventing any general conclusions to be reached on its complex determinism^10^,^12^. The climatic debt in a community results from numerous ecological and anthropogenic constraints such as species migration, tolerance to climate change, species lifespan, evolutionary adaptation, species interaction, environmental stresses, landscape structure and human management^12^,^21^,^22^ (Fig. 1). Assessing the main determinants of the climatic debt is crucial to get a better picture of the vulnerability of biodiversity to climate change, and to validate and improve forecasts of biodiversity changes under the twenty-first century climate change^10^,^12^.

Here we investigate the ecological determinism of the climatic debt reported in French forest herbaceous plant communities (mean debt=1.05 °C, s.d.=1.25 °C)^8^ from a total of 67,289 floristic surveys carried out between 1987 and 2008 (that is, the most recent climate warming period characterized by +1.07 °C compared with 1965–1986, on average)^8^. The climatic debt (hereafter *dT*) was inferred as the difference between the annual mean temperature observed at the date and location of each floristic survey and the temperature bioindicated by its floristic assemblage (that is, the optimal temperature for the local assemblage of herbaceous species)^8^. A large value of the climatic debt means that the composition of plant communities is not shifting towards warm-adapted species as fast as expected from climate warming alone. We first built a conceptual framework of the potential determinants of the climatic debt (Fig. 1). These include the direct effects of the environmental context (that is, baseline conditions and magnitude of change defining the exposure of communities to their abiotic environment) as well as its indirect effects through mechanisms that promote either species' persistence (leading to extinction debt^23^) or migration (leading to immigration credit^23^)^12^,^15^,^16^,^24^. We tested and assessed the contribution and magnitude of 23 abiotic, biotic and anthropogenic variables (see Table 1 and Supplementary Data 1 for a complete list and definition of the variables) by fitting partial least squares (PLS) regressions^25^ on 5,000 different subsamples of floristic observations distant 10 km from each other to limit the effect of spatial autocorrelation in the statistical models^26^. We included several environmental and anthropogenic factors involved in current global changes (climate conditions and changes, soil toxicity and nutrient depletion, natural and anthropogenic disturbances), which are expected to be stressful for plants^27^,^28^ and hence to inflate the climatic debt (Fig. 1). We also included several key factors that favour species' persistence (that is, factors maintaining species survival locally, such as tolerance to climatic stresses, evolutionary adaptation, acclimation and/or phenotypic plasticity, microclimatic buffering, nutrient resources, species' longevity and resource competition), which are expected to inflate the climatic debt by increasing the temporal inertia of floristic assemblages, as well as factors that favour species' migration (that is, factors enhancing species range shifts such as climate niche tracking, habitat connectivity, earliness of reproduction and resource competition), which are expected to mitigate the climatic debt by promoting reshuffling of floristic assemblages (Fig. 1). Lastly, a time effect was introduced in all our statistical models to capture any remaining temporal signal in the climatic debt not accounted by the set of ecological and anthropogenic factors considered in this study (see Methods section for more details).

We show that the climatic debt of the herbaceous plant communities in French forests is determined by a combination of ecological constraints and biotic factors. Warmer baseline conditions predispose plant communities to larger climatic debts, and climate warming exacerbates these lags. Species' persistence mechanisms, such as plant tolerance to climate warming, increase the climatic debt and outweigh the effect of plant migration in the response of plant communities to the current temperature increase. As climate warming should accelerate during the twenty-first century, we raise some doubts about the sustainability of forest plant communities, which are threatened by both future climate conditions probably exceeding species tolerance and the low dispersal ability of forest species leading to an increase of the climatic debt.

## Results

### A multifaceted determinism of the climatic debt

The climatic debt of forest plant communities is the result of a complex determinism that chiefly involves the effects of baseline temperature conditions, species' intrinsic ability to tolerate water and thermal stresses, and climate warming exposure (Fig. 2). Among the 19 abiotic, biotic and anthropogenic variables tested in the PLS0 model, 13 have a significant effect (bootstrap test for difference of slope values to 0: *P*<0.01, *n*=5,000; Supplementary Table 1). Together, they explain 41.4% (CI_95%_: 38.2 to 44.4) of the total variation in the climatic debt, of which 35.7% (CI_95%_: 32.9 to 38.6) can be assigned to factors that amplify the climatic debt and 5.7% (CI_95%_: 4.7 to 6.7) to factors that mitigate it. Both the magnitude of the effect and the proportion of variance explained by the significant factors involved in species' persistence (cumulative standardized slope=0.792, CI_95%_: 0.692 to 0.889; sum of explained variance=10.2%, CI_95%_: 8.6 to 11.9) are greater than those involved in species' migration (cumulative standardized slope=−0.511, CI_95%_: −0.59 to −0.426; sum of explained variance=6.3%, CI_95%_: 5.3 to 7.3; Fig. 2).

### Robustness of the results

We found very consistent results regarding the determinism of the climatic debt captured by the PLS0 model (Fig. 2 and Supplementary Table 1) and PLS1 to PLS4 models (Supplementary Data 2 and Supplementary Figs 1–3). The effects of the abiotic, biotic and anthropogenic factors presented in Fig. 2 have similar directions and magnitude across all the models (Supplementary Tables 1 and 2, and Supplementary Data 2). Hence, our main findings are robust to both changes in the samples and periods considered (the PLS1 to PLS4 models were fitted on a subset of floristic communities observed after 1993, while the PLS0 model was fitted on all the floristic communities observed after 1987) and to the addition of new factors in the set of explanatory variables.

As the set of explanatory variables we used are inferred either from model predictions (for example, baseline climate conditions, climate changes and soil conditions), incomplete sampling (for example, species' tolerance to climatic stresses), remote sensing (for example, habitat connectivity) or from statistics (for example, temporal niche and distribution conservatisms), each variable has an associated error that could bias the estimation of its effect in the model. Therefore, we compared the effects estimated from the PLS0 model with those obtained after consideration of the error associated with each variable^29^ (when the information was available; Supplementary Table 3). We found very similar values, demonstrating that errors associated to explanatory variables are unlikely to alter the determinism of the climatic debt reported in Fig. 2.

Finally, no spatial autocorrelation was observed in the residuals of PLS0 (Fig. 3) to PLS4 models (Supplementary Fig. 4). This result confirms that the estimation of the effect of each abiotic, biotic and anthropogenic factors was not biased by a remaining spatial autocorrelation that was not already accounted for by the set of explanatory variables considered^26^. Moreover, the time effect was not significant in any of our models, meaning that no remaining temporal pattern is present in the model residuals after accounting for ecological and anthropogenic factors. The insignificance of any spatio-temporal effects demonstrates the relevance of the set of explanatory variables investigated, as well as their ability to capture any amplifying or mitigating effects that determine the climatic debt.

### Climate severity increases the climatic debt

The environmental context (baseline conditions and magnitude of change) explains a large proportion (27.3%, CI_95%_: 24.7 to 29.9) of the climatic debt and contributes to amplify it. Warmer baseline conditions predispose communities to the climatic debt, whereas higher climate warming exposure is an aggravating factor that further inflates it (Fig. 2). Forest plant communities are more likely to lag behind climate change in warm regions (Fig. 4 and Supplementary Fig. 5), because the regional species pool does not yet include warmer-adapted species that would require unlikely long-distance migration (for example, from Spain or North Africa) to keep up with climate change (Supplementary Fig. 6a). Moreover, warm areas in France are generally located in lowlands (Supplementary Fig. 6b), where the distance between analog temperature conditions before and after 1987 (median value=35.6 km)^8^ is larger than the migration capacity of perennial plants across a fragmented forest territory^30^,^31^, thus further inflating the climatic debt.

### Species persistence outweighs species migration

Although (rapid^32^) species range shifts are reported in the litterature^24^, our results provide evidence that factors favouring species' persistence (which amplify the extinction debt^23^) are more important than factors favouring species' migration, resulting in a delayed response of forest plant communities (Fig. 2). As expected, all factors favouring species' persistence amplify the climatic debt, with plants' intrinsic ability to tolerate climatic stresses being the most prominent one (Fig. 2). This finding supports recent suggestions that species' sensitivity to climate change is an important component of the climatic and extinction debts^8^,^16^,^18^. Although soil quality is rarely accounted for in climate change studies, we found that the best soil conditions in forests (that is, low acidity and high nitrogen content) amplify the climatic debt, most probably by alleviating part of the climatic constraint on plant development and thus favouring local persistence^33^. Species' longevity also inflated the climatic debt, consistent with the idea that biotic responses of long-lived plants are delayed^11^. Evolutionary adaptation, acclimation and phenotypic plasticity inflated the climatic debt, most probably through thermal niche shifts that allow species to conserve part of their distribution areas occupied before contemporary climate warming. However, their limited impact on the climatic debt supports the idea that adaptation is not the main determinant of the delayed vegetation response to contemporary climate change^3^. This stems from the fact that most forest herbaceous species are perennials (86.7% out of the 760 species studied) that reach reproductive maturity after several years, which limits gene flow and the efficiency of evolutionary adaptation over the short time scale of contemporary climate change^34^. Although microclimatic refugia have often been highlighted as important features allowing species to persist locally as climate changes^35^,^36^, our results suggest that this factor is playing a minor role in comparison with other determinant of the climatic debt. For instance, thermal heterogeneity due to topographic complexity is expected to contribute to species' persistence by buffering climate warming through short-distance escapes (<1 km), but it did not significantly explain the climatic debt of herbaceous plant communities in French forests.

In contrast, factors favouring species' migration mitigate the climatic debt, but their effect is relatively weak (Fig. 2). Thus, migration does not counteract the amplifying effect of climate severity and species' persistence mechanisms. Plant communities that are chiefly composed of species tracking their climatic niche through migration have the lowest climatic debt (Fig. 2). Low age at first reproduction mitigates the climatic debt, because short-lived plants migrate faster^7^,^11^. Moreover, the climatic debt is mitigated by the proximity to the closest site that was suitable in the past. This result shows that the match between the composition of present plant communities and their new climatic conditions depends on past species distributions because of the higher probability of immigration or rescue from already occupied patches^11^,^37^. Surprisingly, the current habitat fragmentation in French forests due to anthropogenic land-use changes (see the mean change value of the temporal change in species habitat aggregation index in Supplementary Data 1), which is expected to impede the migration of warm-adapted plant species^30^, does not significantly amplify the climatic debt of forest plant communities. Finally, species' migration seems to be more efficient in highland forests where the climatic debt is low (Fig. 2). Highland areas offer shorter-distance escapes facing climate warming compared with lowland forests (median distance of temporal isotherms shift=1.1 km upward^8^), which allow forest plants that have limited dispersal abilities^30^ to track their climatic requirements, thus decreasing the climatic debt.

### The effect of plant competition depends on the resource

Competition for resources has been reported to delay or even reverse the expected biotic responses to climate change by favouring the locally established competitive species, thereby increasing the resistance of the recipient community to the establishment of new incoming species^22^,^38^. In agreement with this hypothesis, a stronger competition for soil nitrogen amplifies the climatic debt (Fig. 2), probably by selecting locally established plant species that are better adapted to nutrient stress than to climatic stress. However, competition for water has an opposite effect (Fig. 2), probably because it selects drought-tolerant species that are adapted to the new warmer conditions, thus mitigating the climatic debt. These opposite patterns for nutrient and water resources call for future research investigating the effects of competition for different resources on biotic responses to climate change and question the importance of climatic stress compared with other environmental stresses in predicting biodiversity changes.

### Low impacts of forest disturbances on the climatic debt

Neither spatial fragmentation of species habitat due to land-use changes nor recent silvicultural practices (for example, tree cutting, tree extraction, cleaning brush and forestry machine passage), introduction of exotic trees or any other natural or anthropogenic disturbances (for example, fire, storm, inundation and agricultural activity encompassing livestock grazing and abandonment of agriculture areas) had a significant effect on the climatic debt (Supplementary Fig. 2 and Supplementary Data 2 presenting the results of the PLS2 model). Consistently, the determinism of the climatic debt was similar in both disturbed and undisturbed forests (Supplementary Fig. 3, Supplementary Table 4 and Supplementary Data 2 presenting the results of the PLS3 and PLS4 models). These results challenge recent findings obtained at a smaller scale, which suggest that silvicultural practices and local disturbances are drivers of vegetation changes^39^,^40^, notably by favouring the thermophilization of plant communities^20^. Only light availability and nutrient resources, which are at least partly affected by forest disturbances^20^,^39^, significantly explained the climatic debt (3.1% of its variation, CI_95%_: 2.4 to 4.1; Fig. 2 and Supplementary Table 1). Surprisingly, we found that light availability amplifies the climatic debt. This result contradicts previous findings, suggesting that canopy closure should inflate the climatic debt by providing microclimatic conditions that buffer understory plant responses to warming^19^, while canopy opening, which acts as a strong disturbance, should facilitate the establishment of warm-adapted species, thus deflating the climatic debt^20^. We explicitly tested for this effect in the PLS2 model, but the temperature buffering effect due to canopy cover did not significantly explain the climatic debt of French forest plant communities (Supplementary Fig. 2 and Supplementary Data 2). Our finding suggests two alternative scenarios leading to an increase in the climatic debt: either (i) forest opening (due to natural and/or human-induced disturbances) plays a more important part in changes in community composition than does climate change and, as a consequence, some forest plant communities are reshuffled based on the light stress they experience, or (ii) it increases temperatures inside the forest stand (in addition to climate warming through rising solar radiation on the forest floor) so drastically that the local species pool, which is adapted to cooler climate conditions, cannot cope with them (Supplementary Fig. 7).

The effect of human population density was significant in three of the five models fitted (PLS1, PLS2 and PLS4; Supplementary Figs 1–3). It is interesting to note that it amplifies the climatic debt probably through long-term and persistent human disturbances depauperating the regional species pool^41^. Although our findings about the effects of silvicultural practices, natural and human-mediated disturbances on the climatic debt are challenging, they probably need more investigations before any general conclusion can be drawn. In particular, it would be useful to consider quantitative variables that capture the intensity of forest disturbance and management, which we were unable to include in our models due to a lack of data at the appropriate spatial scale.

## Discussion

The predominance of predisposing (that is, a warm baseline macroclimate) and aggravating (that is, a strong exposure to climate warming) factors and species' persistence mechanisms in the determinism of the current climatic debt question the sustainability of forest plant communities. According to future climate projections (+1.6 to +4.9 °C is predicted by representative concentration pathway scenarios for the period 2091–2100 relative to 1961–1990 on average throughout the French territory)^14^, climate change should further increase the climatic debt and might eventually exceed species tolerance, thereby triggering local extinction events, novel species assemblages and unknown cascading effects on ecosystem functioning^2^. In particular, the sustainability of forest plant communities occurring in warm and dry areas (such as the Mediterranean ecosystem), for which the climatic debt is already large and the pool of species adapted to warmer conditions is limited (Supplementary Fig. 6a), is threatened. Under such a scenario, the seemingly good news that species' persistence mechanisms buffer forests against current climate warming might be detrimental in the long run, making it impossible for plant communities to recover a growing climatic debt due to the accumulation of time lags in the biotic responses to climate warming. Some doubts may be raised about the ability of species dispersal to allow plants to track future climate change and recover the climatic debt. Forest plant communities are mainly composed of perennial species that have delayed seed dispersal^11^, low long-distance dispersal ability in fragmented habitats^30^ and probably shorter seed dispersal than the geographical redistribution of climate conditions (ranging from 0.4 and 137.6 km for a 1.07 °C increase between 1965 and 2008; ref. 8). Current silvicultural practices, which have no effect on the current climatic debt, are unlikely to be efficient at preventing the climatic debt of forest plant communities to rise in the face of the predicted magnitude and velocity of future climate warming. Our results, however, suggest that limiting light stress through clearcut prohibition for instance and regulating human disturbances may hamper the amplification of the climatic debt driven by these additional constraints on forest herbaceous plant communities and, as a consequence, may improve their sustainability under climate warming. Humans will unlikely assist migration of non-commercial plants such as herbaceous species due to its considerable costs in terms of work and funding, but silvicultural practices aiming to reshuffle the composition of tree communities in accordance with climate change could favour the establishment of climate-adapted understory plants through both changes in local environmental conditions induced by trees and interactions between trees and herbaceous plants^42^.

## Methods

### Climatic debt assessment

Our work is based on the Hutchinsonian niche concept^43^, which considers that the growth and persistence of a species is determined by its environmental requirements. Under this concept, it is assumed that climate change drives species range shifts^3^,^7^,^32^ and, as a consequence, leads to the reshuffling of biotic communities. Therefore, species assemblages can be used to infer climatic conditions at a given location and time^8^,^9^, and if biotic communities responded instantaneously to climate change, they would reflect existing climatic conditions. Here we used forest plant communities (observed in 67,289 surveys) to infer temperature conditions over a 22-year period (1987–2008) in France and computed an index of vegetation disequilibrium with temperature. This index is the difference between the annual mean temperature at a given location and year reconstructed from climate models (*CrT* as climatically reconstructed temperature) and the annual mean temperature of the same location and year but inferred from plant assemblages (*FrT* as floristically reconstructed temperature)^8^. A positive difference (that is, *dT*=*CrT*−*FrT*>0) means that the observed reshuffling in the focal plant assemblage is lagging behind climate warming^8^ and thus depicts a climatic debt^9^ for that assemblage.

*CrT* values were extracted from a set of 22 yearly temperature grids (from 1987 to 2008) at a 1 km^2^ resolution across France. All temperature grids were derived through models predicting temperature conditions from topographical, geographical, solar radiations and land-cover variables (*R*^2^=0.93 and root-mean-square deviation (RMSD)=0.56 °C for 13,620 independent temperature observations)^8^. *FrT* values were modelled in a previous study^8^ using a transfer function that combines a weighted averaging PLS regression^44^ (accounting for linear effect) and a Breiman's random forest model^45^ (accounting for nonlinear and interaction effects among species occurrences in residuals of the weighted averaging PLS model) to infer temperatures from plant community composition (presence/absence of herbaceous species). This transfer function was calibrated on a training data set of 2,987 surveys with a minimum of 5 co-occurring species in each survey, all sampled before the study period (1975–1985, that is, before the onset of contemporary climate warming, to not introduce any vegetation response to climate change in the model calibration) and encompassing the most common herbaceous species, to limit misidentification issues of rare species in our data (that is, having at least 5 occurrences; *n*=760 species; listed in the Supplementary Data 3). The underlying assumption of this transfer function is that plants and climate were at equilibrium before the onset of contemporary climate change (that is, 1987) and during the entire period covered by the calibration data set^46^. This assumption was verified by the absence of climatic debt observed in the French forest plant communities between 1965 and 1986 (ref. 8). The transfer function was validated on an independent data set of 5,136 surveys sampled between 1975 and 1985 (*R*^2^=0.83, RMSD=0.97 °C). More details about the computation of the *FrT* values are provided in the Supplementary Methods.

Both *CrT* and *FrT* were extracted and predicted, respectively, at the location and year of each of the 67,289 georeferenced and dated floristic surveys recorded during the period 1987–2008 and stored in three French databases (IGN-IFN^47^, which is a forest inventory database, *n*=54,618 plots; Sophy^48^, which is a phytosociological database, *n*=9,575 plots; and EcoPlant^49^, which is a phytoecological database, *n*=3,096 plots; Supplementary Fig. 8). Based on *CrT* and *FrT*, we computed *dT* for each floristic survey (Supplementary Data 4), yielding an average climatic debt of 1.05 °C (s.d.=1.25 °C)^8^.

### Potential determinants of the climatic debt

**Figure 1:**
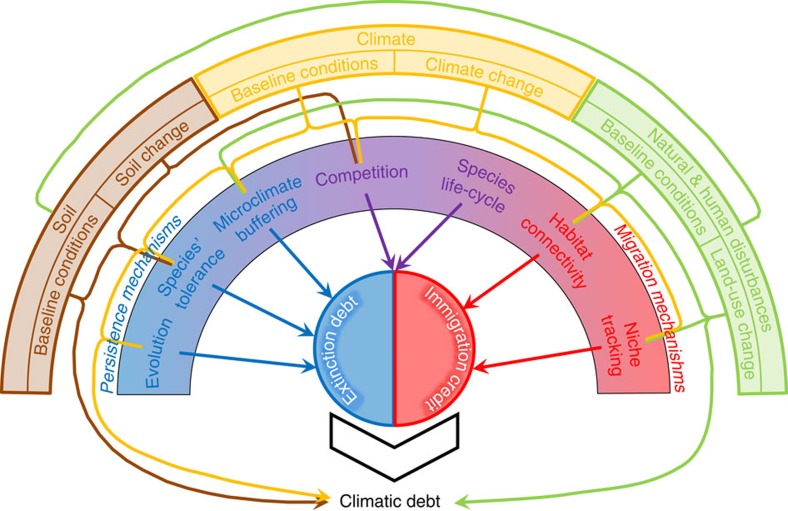
Conceptual framework of the climatic debt determinism. The climatic debt is an integrative measure of the lag in the reorganization of plant communities driven by climate change, but also by the environmental context (that is, baseline conditions and magnitude of change) that interferes with this response. These environmental factors can have an impact on plant communities either directly or indirectly, through biotic mechanisms involved in species' persistence and species' migration, leading to an extinction debt and an immigration credit, respectively, thereby modulating the climatic debt.

**Figure 2:**
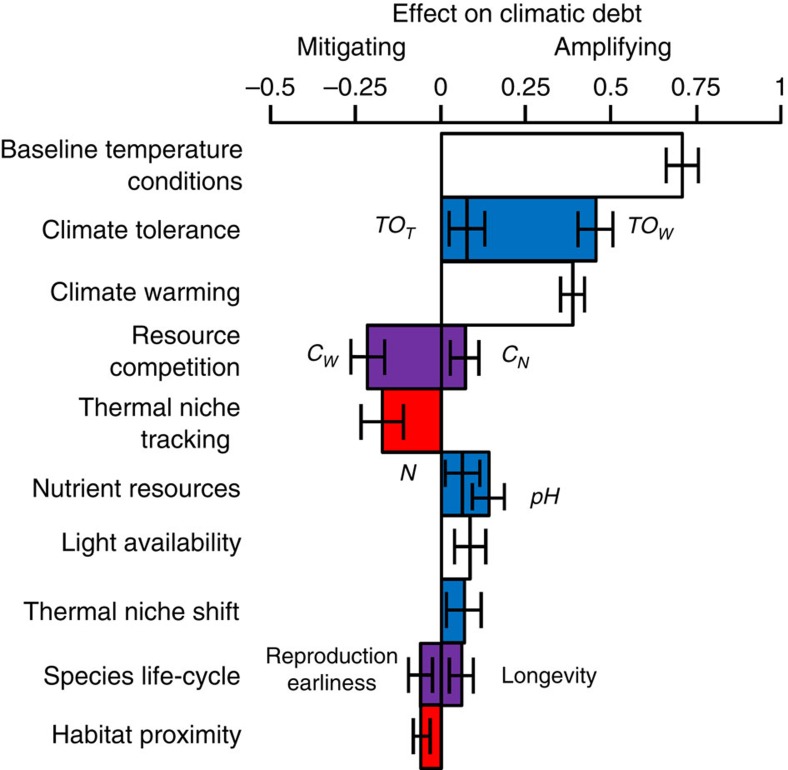
Ecological determinism of the climatic debt in forests. The effect of each variable is measured by the mean slope of the climatic debt observed in French forest herbaceous plant communities between 1987 and 2008 *versus* this variable over the 5,000 subsamples of the PLS0 model. Error bars show 95% confidence intervals. Only significant variables are shown (bootstrap test for difference of slope values to 0: *P*<0.01, *n*=5,000) and classified by categories of factors. Temporal changes in species habitat aggregation, road proximity, human population density, temperature heterogeneity, precipitation change, baseline precipitation conditions and time were not significant (Supplementary Table 1). Negative and positive slopes mean mitigating and amplifying effects, respectively, on the climatic debt. Factors involved in species' persistence and migration are specified in blue and red, respectively; purple depicts factors involved in both species' persistence and migration; white depicts environmental pressures for species. *C*_*N*_, interspecific competition for soil nitrogen; *C*_*W*_, interspecific competition for water; *dHA*, temporal change in species habitat aggregation; *HP*, past species habitat patches' proximity; *N*, soil nitrogen content; *pH*, soil acidity; *TO_T_*, thermal-stress tolerance; *TO_W_*, water-stress tolerance.

**Figure 3:**
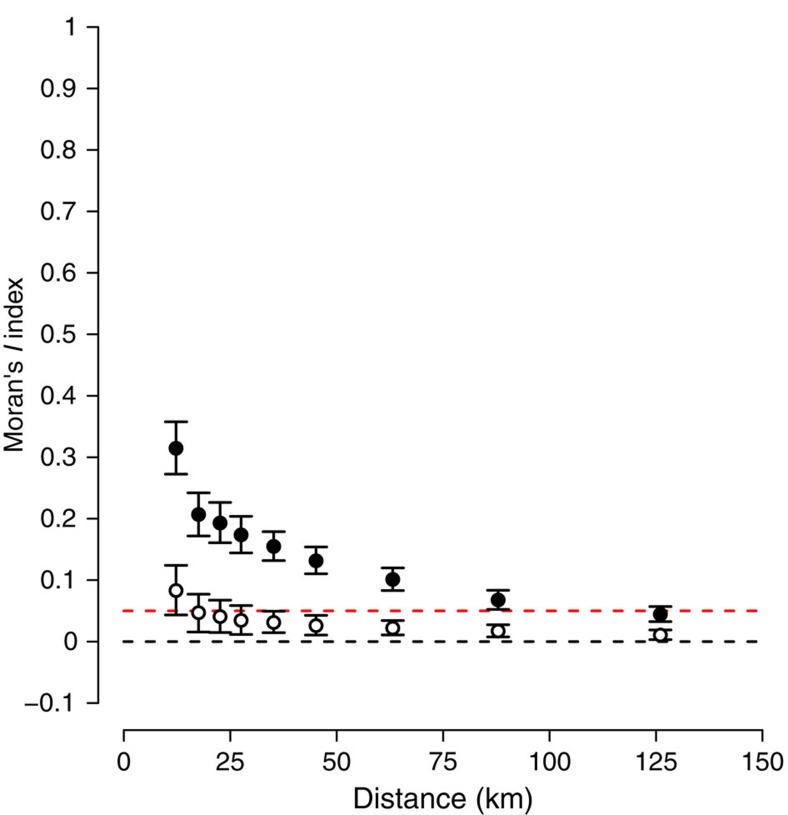
Spatial correlograms computed from the climatic debt and residuals of the PLS0 model. Black and white dots show mean values of the Moran's *I* index computed on the climatic debt and the model residuals (*n*=5,000 bootstraps), respectively. Error bars show 95% confidence intervals. The red dotted line depicts the threshold of spatial autocorrelation significance (that is, *I*=0.05).

**Figure 4:**
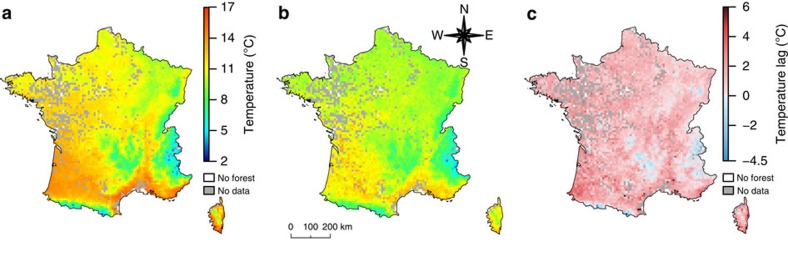
Comparison of the spatial distributions of the observed and bioindicated temperatures. (**a**) Map of the baseline temperature conditions (*CrT*). (**b**) Map of the bioindicated temperatures from the forest herbaceous plant communities (*FrT*). (**c**) Map of the observed lag of temperatures between *CrT* and *FrT* (*dT*). The *dT* variable is computed as the difference between *CrT* and *FrT* in such a way that positive values represent climatic debts in plant communities. The maps show average values in a 10 km grid computed from the 5,000 subsamples used to fit the PLS0 model. The temperature values mapped in **a**,**b** range from 2 to 17 °C across France and is materialized by a colour gradient ranging from blue to red (a detailed colour scale is provided to the right of **a**). The *dT* values mapped in **c** range from −4.5 to 6 °C and is materialized by a colour gradient ranging from blue to red (a detailed colour scale is provided to the right of **c**). White and grey pixels are areas without forest territory and without floristic survey, respectively. The map of the s.d. of *dT* is provided in Supplementary Fig. 5.

As the aim of our study was to assess the main determinants of the climatic debt across the French territory (552,000 km^2^), we computed several abiotic, biotic and anthropogenic variables, which are known or at least suspected to have an impact on the response of forest herbaceous plant communities to climate change both directly, through baseline conditions and magnitude of changes^15^,^16^, and indirectly, through mechanisms involved in species' persistence and migration^16^,^24^ (Fig. 1, Table 1 and Supplementary Data 1).

First, we computed 14 variables depicting baseline environmental conditions and species exposure to environmental changes. Initial climatic conditions (macroclimate) before the onset of contemporary climate change are likely to have an impact on the composition and size of the regional species pool from which plant communities can be reshuffled over a short timescale^37^ and thus are also likely to affect the magnitude of the observed climatic debt. To account for the effect of baseline climatic conditions, we extracted both the annual mean temperature (*T*; RMSD=0.21 °C for 180 temperature observations) and the annual precipitation (*P*; RMSD=66 mm for 429 precipitation observations) over the 1965–1986 baseline period at the location of each of the 67,289 surveys. As described above for temperature data, annual precipitations were extracted from a set of 22 grids (from 1965 to 1986; 1 km^2^ resolution) across France. All grids were derived through models predicting precipitations from geographical and physiographical variables^50^ (Supplementary Fig. 9). As a higher climate change velocity decreases species' intrinsic abilities to respond synchronously to climate change^13^, plant assemblages are then more likely to lag behind climate change^8^,^9^. To account for climate change exposure on the observed climatic debt, we computed changes in annual mean temperatures (*TC*; RMSD=0.04 °C for 3,960 observations) and annual precipitations (*PC*; RMSD=145 mm for 9,438 observations) between the year of the floristic observations and the 1965–1986 baseline period.

Local environmental conditions in a forest stand also have an impact on understory plant community^39^,^51^. Soil conditions drive floristic changes, both directly^27^,^51^ (for example, when a nutrient stress adds to the climatic one) and indirectly (for example, when the climatic constraint is alleviated by good soil conditions)^33^. In the absence of direct soil observations over the 67,289 surveys, soil pH (*pH*) and C:N ratio (*N*) were inferred from a bioindication model using the floristic assemblage to predict soil pH^51^ and C:N ratio^52^ (RMSD=0.9 and 3.1 for 254 independent pH and C:N ratio observations, respectively; Supplementary Fig. 10). It is noteworthy that in our analysis, we used the inverse of the C:N ratio, to depict an increasing gradient of soil nitrogen content. Light availability, driven by natural disturbances, forest development and management, is playing a dual effect on the composition of floristic assemblages by disturbing resident understory plant communities as light increases sharply^20^,^40^ and buffering part of the climate change as the canopy cover increases^19^. As a consequence, it can modulate the climatic debt of forest plant communities. The direct impact of light availability on the composition of understory plant communities (*L*) was inferred by computing the average value of the L-Ellenberg index^53^ (ranging from 1 to 9, that is, from shade-tolerant to light-adapted species) for all co-occurring species in each survey. It is noteworthy that although several variables (*FrT*, *L*, *pH* and *N*) were computed from bioindication methods, they were largely uncorrelated (*R*^2^<0.1; Supplementary Data 5), demonstrating no circularity issues between these indices. The plant species contributing to bioindicate each environmental condition differ due to differences in climate, light and edaphic requirements among species, which limit the risk of accounting for confounding environmental conditions in predictions. Bioindication methods allow inference of local environmental conditions that account for both baseline conditions and environmental changes^50^,^51^. The temperature buffering effect due to canopy cover (*TBUF*) was computed from the microclim model^54^ as the difference between the temperatures perceived on the forest floor considering and not considering the recorded canopy cover. The microclim model was run using forest stand characteristics (canopy cover and height) and interpolated climate conditions at the date and location of each floristic relevé (details on the method are provided in Supplementary Methods, Supplementary Fig. 11 and Supplementary Table 5). Similarly, temperature heterogeneity within a 1 km^2^ macroclimatic unit has the potential to partly buffer the species exposure to climate change by providing short-distance microclimatic refugia that enhance the local persistence of species despite unfavourable regional climate conditions^34^,^35^. To account for this effect, we computed temperature heterogeneity within each 1 km^2^ unit (that is, the spatial resolution of all climatic grids we used; Supplementary Fig. 12) by using a finer-resolution temperature grid (2,500 m^2^; *R*^2^=0.93 and RMSD=0.58 °C for 13,620 independent temperature observations; Supplementary Fig. 13)^50^. It is noteworthy that we did not use this finer-resolution temperature grid when computing the climatic debt because of the 500 m uncertainty in the geographic location of some floristic surveys.

Finally, we used five more variables to account for the potential impact of anthropogenic and natural disturbances. Three qualitative variables (coded 1 or 0) informing for the presence/absence of recent silvicultural practices (*SILVP*; for example, observation of tree cutting, tree extraction, cleaning brush and forestry machine track), the presence/absence of human-mediated and natural disturbances (*DISTURB*; for example, observation of fire, storm, inundation and agricultural activity encompassing livestock grazing and abandonment of agriculture areas) and the presence/absence of exotic trees (*EXOT*; considering 24 species defined by the Inventaire National du Patrimoine Naturel and listed in Supplementary Table 6) were extracted from floristic database. We also computed proximity to road as the minimum distance between each floristic inventory plot and the French road network (that is, a spatial layer downloaded from the GeoFabrik website^55^ and containing all linear road structures from highways to forest tracks). Finally, we extracted the sum of the human population density (*HPD*; assessed in 2009; ref. 56) in a radius of 10 km around each floristic inventory plot. Both variables are used to infer the human impact on biodiversity^41^ through forest management^40^ and/or assisted migration and invasion^57^, as well as the impact of natural disturbances^20^,^58^.

The *TBUF*, *SILVP*, *DISTURB* and *EXOT* variables were computed or extracted from a subset of 45,806 floristic surveys belonging to the IGN-IFN^47^ database over the 1993–2008 period for which the information was available.

Second, we computed three variables depicting important biotic mechanisms involved in species' persistence. Not only baseline environmental conditions and exposure to environmental changes but also species' intrinsic ability to tolerate environmental changes are likely to explain the observed delayed response of plant communities to climate change^16^,^18^. For instance, plant assemblages with a large proportion of species that are relatively insensitive to climate (that is, with a wide climatic-niche breadth) are more likely to lag behind climate change than plant assemblages with a large proportion of species that are relatively sensitive to climate (that is, with a narrow climatic-niche breadth). To test for such an effect, we first inferred the temperature and precipitation niche breadths (*cf*. thermal- and water-stress tolerances, respectively) for each of the 760 herbaceous plant species mentioned earlier. For a given species, we computed the ranges between minimum and maximum annual mean temperature and annual precipitation where the focal species occurred between 1961 and 1986 (*n*=14,954 floristic surveys from Sophy and EcoPlant databases; Supplementary Data 3). The 1961–1986 period was used here for its relative stability in terms of macroclimatic conditions, thus allowing us to compute robust climatic niche breadths considering the equilibrium observed between plants and climate^8^, and to not account for any climatic niche shift in response to climate change due to evolutionary adaptation, acclimation and/or phenotypic plasticity that we want to test explicitly the effect in our framework (see below). Both measures were then averaged across all species co-occurring within a given plant assemblage to get two community mean values characterizing the focal plant assemblage's tolerance to thermal (*TO*_*T*_) and water (*TO*_*W*_) stresses.

Species may also persist locally through evolutionary adaptation, acclimation and/or phenotypic plasticity by shifting their ecological requirements (that is, niche shifts), to survive to the new climatic conditions^59^,^60^. The extreme case of complete adaptation, acclimation and/or plasticity to the new climatic conditions means distribution conservatism over time (*DC*). Owing to spatio-temporal variability in our data, we cannot assess *DC* directly (Supplementary Fig. 14). For each species, we first assessed the past thermal niche (*N*_0_) by extracting annual mean temperature conditions during 1965–1986 at all localities where the focal species occurred during 1965–1986 (from a total set of 13,992 floristic observations). Second, we inferred the expected thermal niche (*N*_exp_) under the assumption that the focal species did not change its spatial distribution between 1965–1986 and 1987–2008. We computed *N*_exp_ by extracting annual mean temperature conditions during 1987–2008 at all localities where the focal species occurred during 1965–1986 (from the same set of 13,992 floristic observations). Third, we computed the thermal niche overlap (*D*_ref_) between *N*_0_ and *N*_exp_ using the Schoener's *D* index^61^, which ranges from 0 to 1 (that is, no overlap to perfect overlap). Fourth, we assessed the observed thermal niche (*N*_obs_) by sampling the annual mean temperature conditions during 1987–2008 among all localities where the focal species occurred during 1987–2008 (from a total set of 131,657 floristic observations) that it matches *N*_exp_. Such sampling strategy allows comparing thermal niches over time with similar accuracy. Without this step, artificial thermal niche shifts due to differences in the number and distribution of species occurrences between both studied time periods can mask the effect of evolutionary adaptation, acclimation and/or phenotypic plasticity. Fifth, we computed the thermal niche overlap (*D*_obs_) between *N*_0_ and *N*_obs_ using Schoener's *D* index^61^. Finally, we computed an index of species' distribution conservatism over time (*DC*_sp_) as the ratio between *D*_obs_ and *D*_ref_. It is noteworthy that *D*_obs_ never exceeds *D*_ref_ and at best equals *D*_ref_, and thus *DC*_sp_ cannot exceed 1. Distribution conservatism for a species is maximum and so is evolutionary adaptation, acclimation and/or phenotypic plasticity, when *DC*_sp_ reaches 1 (Supplementary Data 3). To compute *DC*_sp_ at the community level (*DC*), we averaged *DC*_sp_ values across all species co-occurring within a given community.

Third, we computed three variables depicting important biotic mechanisms involved in species' migration. Poleward and upward range shifts^3^,^7^ are important biotic responses to climate warming. Climate niche tracking underlies such migrations, whose efficiency is modulated by species' dispersal abilities and habitat fragmentation. A perfect climate niche tracking implies both unlimited seed dispersal (allowing species to migrate towards newly suitable climate conditions as fast as climate warms) and immediate local extinction in the newly unsuitable climate conditions, which lead to niche conservatism over time (*NC*). As for *DC*, *NC* cannot be computed directly due to spatio-temporal variations in our data and thus we also had to assess first *N*_0_, *N*_exp_ and *N*_obs_ (Supplementary Fig. 15). Although we used the exact same values of *N*_0_ for both *DC* and *NC*, the values for *N*_exp_ and *N*_obs_ were here computed differently. This time, *N*_exp_ represents the expected thermal niche under the assumption that the focal species did not shift its thermal niche between 1965–1986 and 1987–2008. We computed *N*_exp_ by sampling annual mean temperature conditions during 1987–2008 among the set of recent floristic surveys (*n*=131,657 between 1987 and 2008), irrespective of the presence or the absence of the focal species, to reconstruct *N*_0_. As previously explained for *DC*, such sampling strategy allows limiting artificial thermal niche shifts. Again, we computed the baseline overlap (*D*_ref_) by comparing *N*_0_ and *N*_exp_ using the Schoener's *D* index^61^. Regarding *N*_obs_, we sampled annual mean temperature conditions during 1987–2008 among all localities where the focal species occurred during 1987–2008 (from 131,657 floristic surveys) so that it matches *N*_0_. Again, we computed the observed overlap (*D*_ob*s*_) between *N*_0_ and *N*_obs_ using Schoener's *D* index^61^. Finally, we computed an index of species' thermal niche conservatism over time (*NC*_sp_) as the ratio between *D*_obs_ and *D*_ref_. It is noteworthy that *D*_obs_ never exceeds *D*_ref_ and at best equals *D*_ref_, and thus *NC*_sp_ cannot exceed 1. Thermal niche tracking over time is perfect and thus species' range shifts are synchronous with climate warming, when *NC*_sp_ reaches 1 (Supplementary Data 3). To compute *NC*_sp_ at the community level (*NC*), we averaged *NC*_sp_ values across all species co-occurring within a given community.

To account for the species-specific effect of habitat connectivity on migration processes, we computed at the species level two variables representative of habitat proximity and habitat change. Species' potential habitat was delineated by the forest areas where temperature conditions are comprised between the lower and upper thermal limits of its thermal tolerance (*cf*. *TO*_*T*_). Past and current potential habitats for each of the 760 studied forest plant species were mapped across the French territory at 1 km^2^ resolution based on past (1965–1986) and current (1987–2008) annual mean temperature conditions, as well as past and current forest covers defined by 1990 and 2000 CORINE Land Cover grids, respectively. French forest areas were determined by merging coniferous, deciduous and mixed forest into one single ‘forest' category. Based on these species-specific sets of two grid layers depicting past and current potential habitats, we computed, for each species separately, past habitat proximity (*HP*) as the minimum distance between all localities where the focal species occur during 1987–2008 and its past potential habitat. This distance represents the proximity to past potential sources of species immigration likely to affect the current plant distribution and community composition^37^. In our analyses, we used the inverse of this minimum distance, to obtain an increasing gradient of *HP*. We also computed an index of temporal change in the aggregation of each species habitat (*dHA*) by computing the difference between its past and current potential habitat aggregation^62^ within a 10 km radius around each floristic survey where the focal species occur during 1987–2008. This 10 km radius was used to only consider changes in habitat aggregation close to where the species occurs, which could affect the most community assembly. Negative and positive values of this index mean disaggregation and aggregation of the species' habitat, respectively. This index is important to consider, because it reflects habitat cohesion and habitat gain or loss driven by both climate change and human activities, which are known to affect plant population dynamics and species migration. Both indices were then averaged across all species co-occurring in a given plant assemblage.

Fourth, we computed three variables depicting important biotic mechanisms involved in species' persistence and migration. Longevity (*LG*) is an important trait that allows species to persist locally in the form of remnant populations despite environmental conditions are deteriorating, and thus that contributes to the climatic debt^11^. In contrast, a shorter lifespan means that plants reach reproduction maturity earlier, leading to faster seed dispersal and gene flows improving species migration and evolutionary adaptation capacities^59^. Information about species longevity for each plant species were collected from the LEDA database^63^ (a database of life-history traits of the Northwest European flora; Supplementary Data 3) and were averaged across all species co-occurring in each plant assemblage.

Resource competition is also expected to affect the responses of species and communities to climate change^22^, because it is an important driver of community assembly^37^. In the absence of resource competition measure in our data, we estimated resource competition among co-occurring species composing forest plant communities. We assumed that species niche differentiation observed in forest plant communities is a good proxy of resource partitioning directly linked to the magnitude of competition^64^. We computed two indices of resource competition: one for water competition (*C*_*W*_), which is directly linked to climate change, and the other for soil nutrient competition (*C*_*N*_), which can be considered as a factor competing with climate change effects. The indices were obtained from the same method, by first computing pairwise niche differentiation along annual precipitation and soil C:N ratio gradients among the 760 studied plant species (niche differentiation*=*1−*D*, with *D* is Schoener's *D* index^61^ measuring the niche overlap), and then by averaging niche differentiation values among the species co-ocurring in each of the 67,289 forest communities investigated (Supplementary Fig. 16). We considered that a high niche differentiation among co-occurring species (*C*_*W*_ or *C*_*N*_ values close to 1) depicts a high resource partitioning probably driven by a strong resource competition in a plant community, whereas a low niche differentiation means that species with close requirements can co-exist locally, demonstrating a low structuring effect of resource competition on plant community composition^64^. We note here that random community assembly could challenge our interpretation of species niche differentiation. We tested for that alternative scenario by comparing for each plant community, a random distribution based on 2,500 *C*_*N*_ and *C*_*W*_ values (computed from random community assembly selected among the regional species pool), with the observed *C*_*N*_ and *C*_*W*_ values, respectively. The regional species pool is defined as the set of plant species found within a 10 km radius around the focal community and is considered as the set of species from which the focal community has been assembled in the field. We showed that 56.8 and 90% of forest plant communities with low (*C*_*W*_ and *C*_*N*_<0.65; *n*=1,034 plots) and high (*C*_*W*_ and *C*_*N*_>0.85; *n*=4,079 plots) species niche differentiations, respectively, are not driven by random assemblage of the regional species pool (Supplementary Fig. 17). It means that strong ecological processes have structured forest plant communities in our data, such as resource competition selecting species with the highest probability to co-exist in a community (that is, species with similar to different requirements as competition increases in the community). Such a result validates our use of *C*_*W*_ and *C*_*N*_ indices as a proxy of resource competition.

### Analytical approach

The effects of all the abiotic, biotic and anthropogenic variables (presented above; see Table 1 and Supplementary Data 1) on the climatic debt were tested and assessed simultaneously in a multivariate analytical model. We used PLS regression, which is a robust method for data analysis, especially when the effects of a great number of explanatory variables are investigated^25^. Basically, PLS regression follows an algorithm searching for the linear combination of explanatory variables (that is, principal components) fitted successively under the orthogonality constraint that maximizes the explanation of the variation in the *Y*-variable (that is, the climatic debt in our case). Compared with a simple linear model, PLS regression has the advantage to provide both (i) an unbiased coefficient estimation even in the presence of multi-collinearity among explanatory variables^25^ and (ii) a robust statistical framework to perform variation partitioning analysis of the *Y*-variable^65^.

The first PLS regression model (PLS0) that was fitted is:


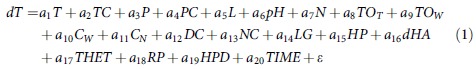


where *a*_1,...,20_ are the estimated coefficients, which we used to quantify the magnitude of the effect of each variable (the definition of variables' abbreviation is provided in Table 1 and in Supplementary Data 1) and *ɛ* denotes the residuals. All variables were centred and reduced to allow parameter comparison.

Four other PLS regressions were fitted from a subset of 45,806 floristic observations sampled between 1993 and 2008. We ran these four extra models, to improve our consideration of the impact of anthropogenic and natural forest disturbances on the climatic debt. First, we fitted a model (PLS1) accounting for the exact same set of explanatory variables included in PLS0 (see equation (1) above). By doing so, we tested for the impact of using a different set of floristic observations covering a slightly different period. Second, we fitted a PLS regression (PLS2) in which the *TBUF*, *SILVP*, *DISTURB* and the *EXOT* variables were tested:


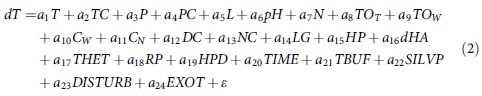


where *a*_1,...,24_ are the estimated coefficients (the definition of variables' abbreviation is provided in Table 1 and in Supplementary Data 1) and *ɛ* denotes the residuals. All variables were centred and reduced to allow parameter comparison.

Third, we fitted two models based on the exact same set of explanatory variables included in PLS0 (see equation (1)) but distinguishing between disturbed (PLS3; *n*=17,954 plots) and undisturbed (PLS4; *n*=27,852 plots) forests. The disturbed and undisturbed sets of floristic observations were determined by the presence/absence of any of the following perturbations: *SILVP*, *DISTURB* or *EXOT*. These two models allowed us to test whether anthropogenic and natural disturbances can modify the ecological determinism of the climatic debt observed in forest plant communities.

All PLS regressions were adjusted using a sub-sampling approach (*n*=5,000), to compute robust and accurate parameter estimation and uncertainty. Subsamples were composed of a set of floristic observations selected randomly (with no replacement) and distant 10 km from each other. Such a distance criterion aims to limit the effect of spatial autocorrelation in the model. Indeed, spatial autocorrelation is known to bias model outputs when it occurs in the model residuals, notably by inverting the relationships captured by the modelling method^26^. To check for potential spatial autocorrelation remaining in the model, we computed the spatial correlograms on residuals of each model using the Moran's *I* index^66^. The numbers of floristic observations selected in each subsample are 2,843, 2,725, 2,725, 2,423 and 2,247 observations for PLS0, PLS1, PLS2, PLS3 and PLS4, respectively, due to the different set of data considered for each of these models. Floristic observations in each of the 5,000 subsamples were weighted in the PLS regressions by the inverse of the total number of observations per year, to correct for an unbalanced temporal distribution of samples. This correction was required in our case, because we wanted to assess the determinism of the climatic debt in a way that is representative of the temporal changes in French forest plant communities between 1987 and 2008. Next, for each subsample, we fitted a PLS regression on the maximum number of principal components (19 for the PLS0, PLS1, PLS3 and PLS4 models, and 23 for the PLS2 model). We investigated the contribution of each principal component to the climatic debt, to determine the number of principal components to be retained in the PLS regression, that is, the set of principal components that summarize significant information for the explanation of the climatic debt. To do this, we fitted a linear model linking the climatic debt to the scores of each observation projected on the 19 or 23 principal components and tested for the significance of the slopes in Student's *t*-test (*P*<0.0025 for PLS0, 1, 3 and 4 and *P*<0.0021 for PLS2, that is, a standard *α* threshold of 0.05 divided by the number of principal components tested simultaneously as a Bonferroni statistical correction). Next, the coefficient values (*a*_1,...,24_), coefficient of determination (*R*^2^), predictions and prediction errors were computed from the significant principal components of the PLS models. We tested the significance of the coefficient values by comparing the distribution of the 5,000 coefficient values with 0. We considered that significant negative and positive coefficient values have at least 99% of the 5,000 coefficients values less and more than 0 (that is, a bootstrap test with a threshold *α*=0.01), respectively. We used this method, because the significance of the coefficients cannot be tested using a Student's *t*-test as in a linear model due to the impossibility of computing the s.e. of the coefficient in PLS regression^67^. Finally, we computed mean and 95% confidence interval (ranging from the percentiles 2.5 and 97.5%) of the coefficient and *R*^2^ values from the 5,000 subsamples.

We note here that circularity issues were not observed between the climatic debt (*dT*) and the baseline temperature conditions (*T*; annual temperature average between 1965 and 1986) and, as a consequence, did not affect the adjustments of PLS regressions (see Supplementary Note 1 for a detailed explanation and Supplementary Figs 18–21). As both PLS regression is a robust modelling method facing multi-collinearity^25^ and explanatory variables are weakly correlated (*R*^2^<0.381; Supplementary Data 5), multi-collinearity issue is unlikely altering our results.

All the variables were computed and all the analysis were conducted in the *R* freeware^68^. The aggregation index was computed using the *SDMTools*^69^ R package and the PLS regressions were fitted using the *plsRglm*^70^ R package.

### Data availability

The floristic data of the IGN-IFN database that support the findings of this study are available from the French National Forest Inventory website (http://inventaire-forestier.ign.fr/spip/spip.php?article532). The floristic data of the EcoPlant and Sophy databases that support the findings of this study are available from J.-C.G. and P.d.R., respectively, but restrictions apply to the availability of these data, which were used under license for the current study, and so are not publicly available. Data are however available from the authors upon reasonable request and with permission of J.-C.G. and P.d.R. The human population density data that support the findings of this study are available from the Institut National de la Statistique et des Etudes Economiques website (http://www.insee.fr/fr/themes/detail.asp?reg_id=0&ref_id=donnees-carroyees&page=donnees-detaillees/donnees-carroyees/donnees-carroyees-km.htm). The road network data that support the findings of this study are available from the GeoFabrik website (http://download.geofabrik.de/). The land-use data that support the findings of this study are available from the Copernicus Land Monitoring Services website (http://land.copernicus.eu/pan-european/corine-land-cover). The climate data that support the findings of this study are available from R.B. but restrictions apply to the availability of these data, which were used under license for the current study, and so are not publicly available. Data are however available from the authors upon reasonable request and with permission of R.B. The LEDA database used to determine species' longevity that supports the findings of this study are available from the Institute for Biology and Environmental Sciences website (http://www.uni-oldenburg.de/en/landeco/research/projects/LEDA/Data%20Files/). *L*, *TO_T_*, *TO_W_*, *LG*, *NC* and *DC* values are provided for each species in Supplementary Data 3. The *dT* values of each floristic survey that support the findings are included in Supplementary Data 4. The authors declare that all other relevant data supporting the findings of this study are available on request.

## Additional information

**How to cite this article:** Bertrand, R. *et al*. Ecological constraints increase the climatic debt in forests. *Nat. Commun.* 7:12643 doi: 10.1038/ncomms12643 (2016).

## Supplementary Material

Supplementary Information Supplementary Figures 1-21, Supplementary Tables 1-6, Supplementary Note 1, Supplementary Methods and Supplementary References

Supplementary Data 1 Definition and numerical description of the 23 ecological factors tested in the models. Each variable was classified among the three main determinants of the climatic debt, i.e. baseline conditions and global changes, species' persistence mechanisms, and species' migration mechanisms. More details about the variables and their computation are provided in the Methods section.

Supplementary Data 2 Statistics of the parameters fitted in the PLS1 to PLS4 models. Average values and 95% confidence intervals (CI_95%_) of R^2^ and slope values were computed from the 5,000 bootstrapped PLS models. The p-values show the results of the bootstrap test for difference of slope values to 0. The significant factors are specified by bold p-values (i.e. p-value < 0.01).

Supplementary Data 3 List of the 760 plant species considered to study the climatic debt in forest plant communities. Number of occurrences in the database (*n*), as well as values of both L-Ellenberg index (*L*), thermal (*TO_T_*; °C) and hydric tolerances (*TO_W_*; mm), species longevity (*LG*, year), and temporal niche (*NC*) and distribution conservatism indices (*DC*) are also provided for each species.

Supplementary Data 4 Lag values between temperature observations and bioindicated temperatures computed for each floristic survey (*n* = 67,289). A positive temperature lag (i.e. *dT* > 0; expressed in Celsius degrees) means a climatic debt in the given forest understory plant communities. Geographical coordinates (*latitude* and *longitude* fields expressed in decimal degrees) is provided with a random uncertainty ranging from 1 to 10 km (according the rules fixed by institutes providing data). Other information are an arbitrary identification number of the surveys (*ID*) and the year of the observation (*year*).

Supplementary Data 5 Correlation matrix of the explanatory variables. The coefficient of determination (R^2^) is used (rather than the correlation value) in order to show the part of variance shared between each variable which is a better statistics to assess multi-collinearity in a set of variables.

## Figures and Tables

**Table 1:**
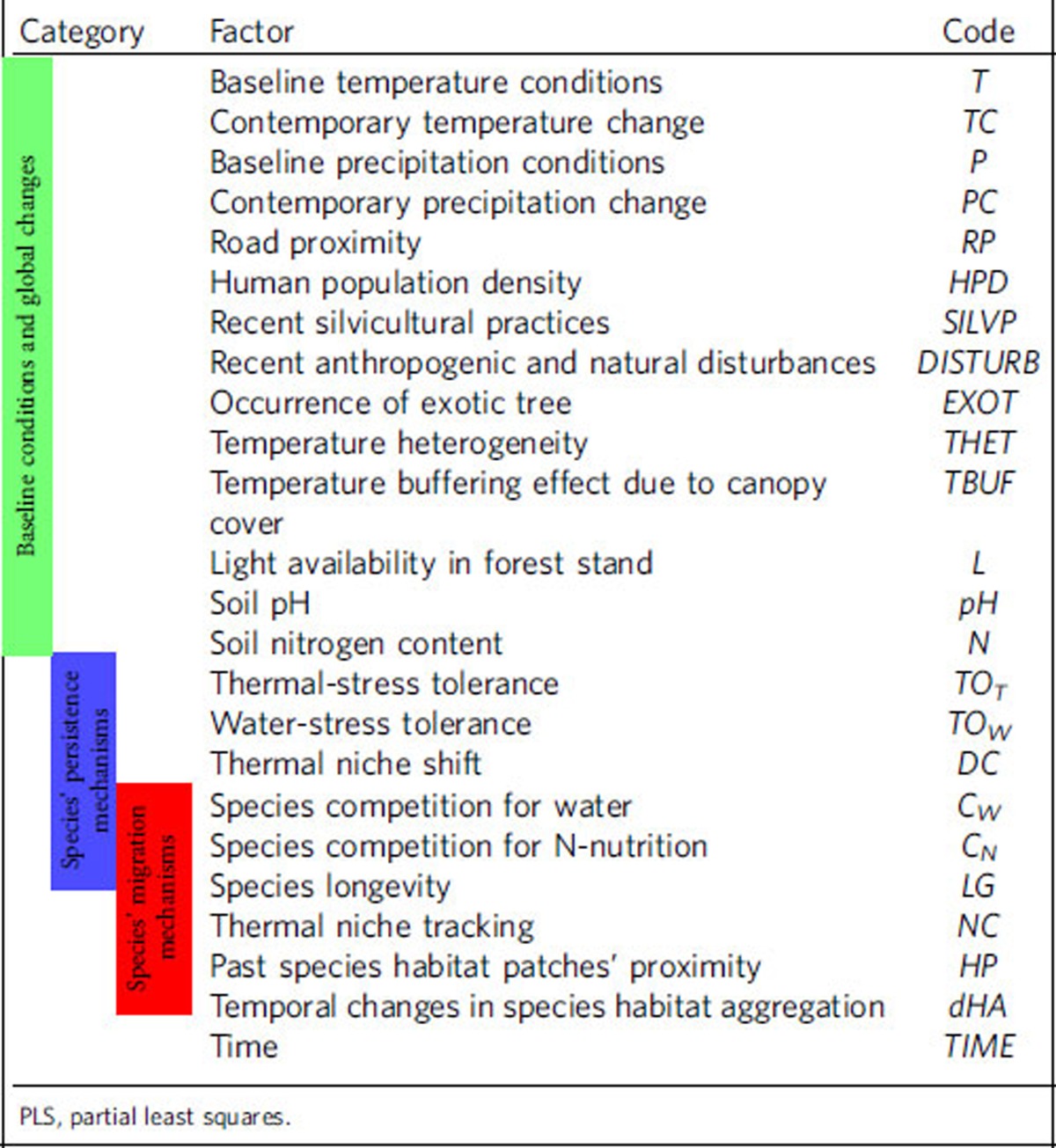
List of the variables tested in the PLS models.
