# Supplementary material for: Ecological constraints increase the climatic debt in forests
Source: Nat Commun. 2016 Aug 26;7:12643. doi: 10.1038/ncomms12643 (PMC5007460; doi:10.1038/ncomms12643)
Supplement: Supplementary Information — Supplementary Figures 1-21, Supplementary Tables 1-6, Supplementary Note 1, Supplementary Methods and Supplementary References [file ncomms12643-s1.pdf]

Supplementary Figures

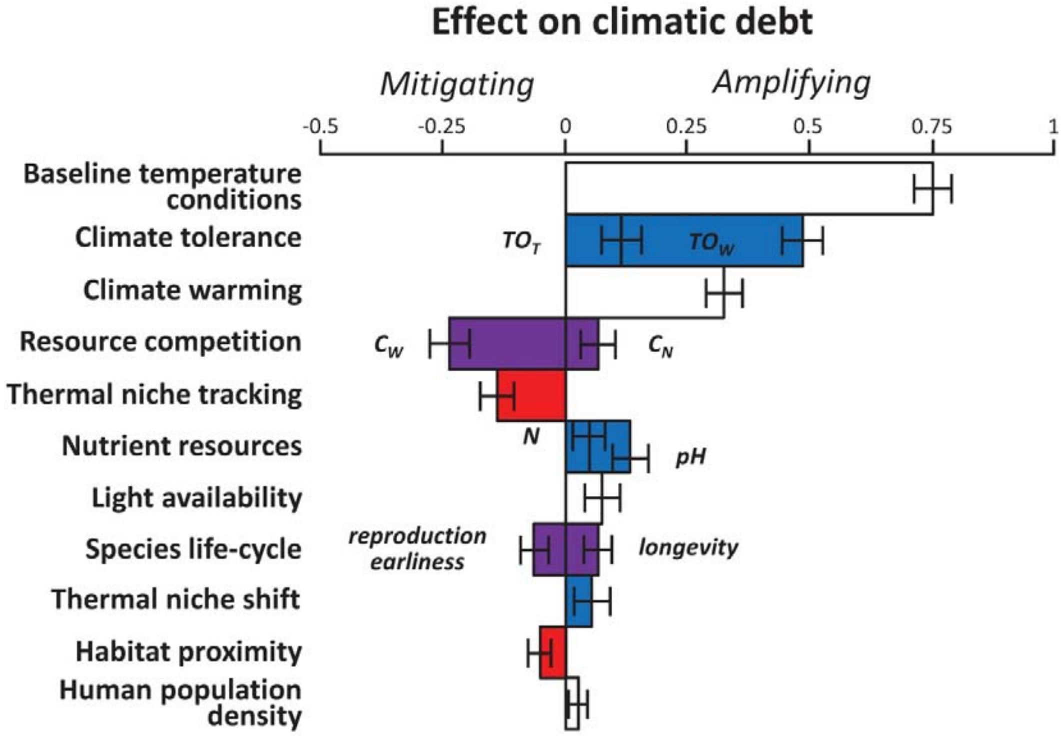

Supplementary Figure 1: Ecological determinism of the climatic debt captured by the PLS1 model. PLS1 is fitted from the same set of explanatory variables than PLS0 but on a subsample of 45,806 floristic observations (see Methods section in the main text). The effect of each variable is measured by the mean slope of the climatic debt *versus* this variable over the 5,000 subsamples of the PLS1 model. Error bars show their 95% confidence intervals. Only significant variables are shown (bootstrap test for difference of slope values to 0: $P < 0.01$, $n = 5,000$), and classified by categories of factors. Temperature heterogeneity, temporal change in species habitat aggregation, road proximity, precipitation change, baseline precipitation conditions and time were not significant (Supplementary Data 2). Factors involved in species' persistence and migration are specified in blue and red, respectively; purple depicts factors involved in both species' persistence and migration; white depicts environmental pressures for species. P = baseline precipitation conditions; T = baseline temperature conditions; TO_T = thermal-stress tolerance; TO_W = water-stress tolerance; C_N = interspecific competition for soil nitrogen; C_W = interspecific competition for water; N = soil nitrogen content; pH =

soil acidity.

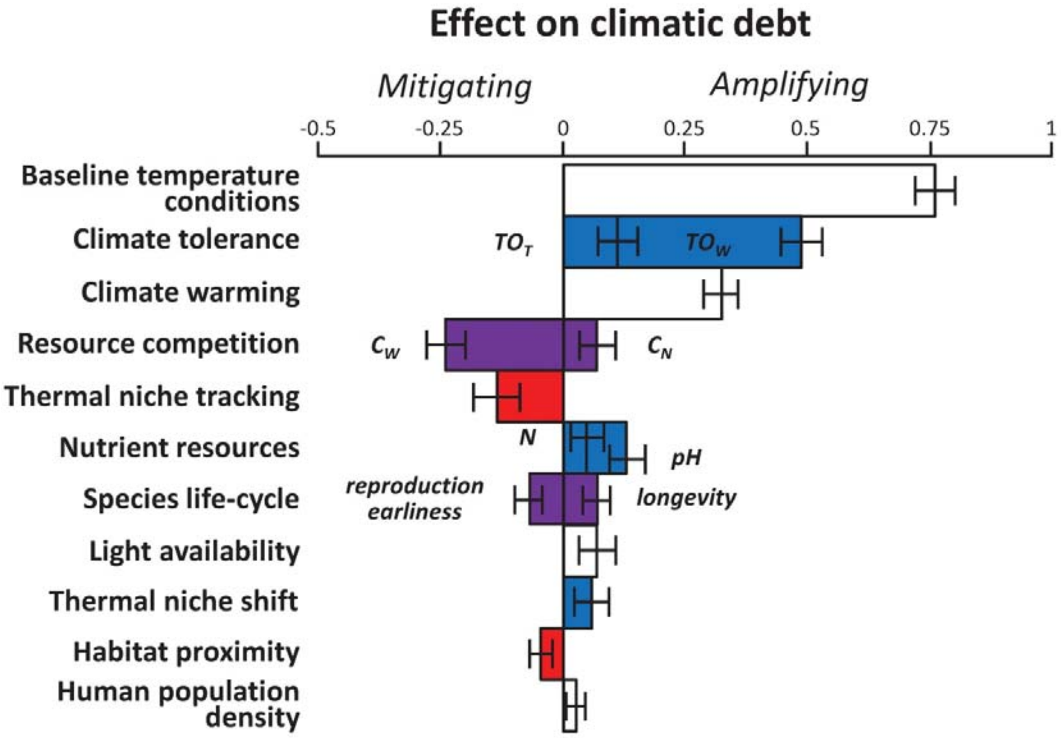

Supplementary Figure 2: Ecological determinism of the climatic debt captured by the PLS2 model. PLS2 is fitted on a subsample of 45,806 floristic observations from the same set of explanatory variables than PLS0 plus four new variables (temperature buffering effect of forest canopy, silvicultural practices, anthropogenic and/or natural disturbances, the presence of exotic tree species; see Methods section in the main text). The effect of each variable is measured by the mean slope of the climatic debt *versus* this variable over the 5,000 subsamples of the PLS2 model. Error bars show their 95% confidence intervals. Only significant variables are shown (bootstrap test for difference of slope values to 0: $P < 0.01$, $n = 5{,}000$), and classified by categories of factors. Temperature buffering effect of forest canopy, forest management, anthropogenic and/or natural disturbances, the presence of exotic tree species, temperature heterogeneity, temporal change in species habitat aggregation, road proximity, precipitation change, baseline precipitation conditions and time were not significant (Supplementary Data 2). Factors involved in species' persistence and migration are specified in blue and red, respectively; purple depicts factors involved in both species' persistence and migration; white depicts environmental pressures for species. *P* = baseline precipitation conditions; *T* = baseline temperature

conditions; TO_T = thermal-stress tolerance; TO_W = water-stress tolerance; C_N = interspecific competition for soil nitrogen; C_W = interspecific competition for water; N = soil nitrogen content; pH = soil acidity.

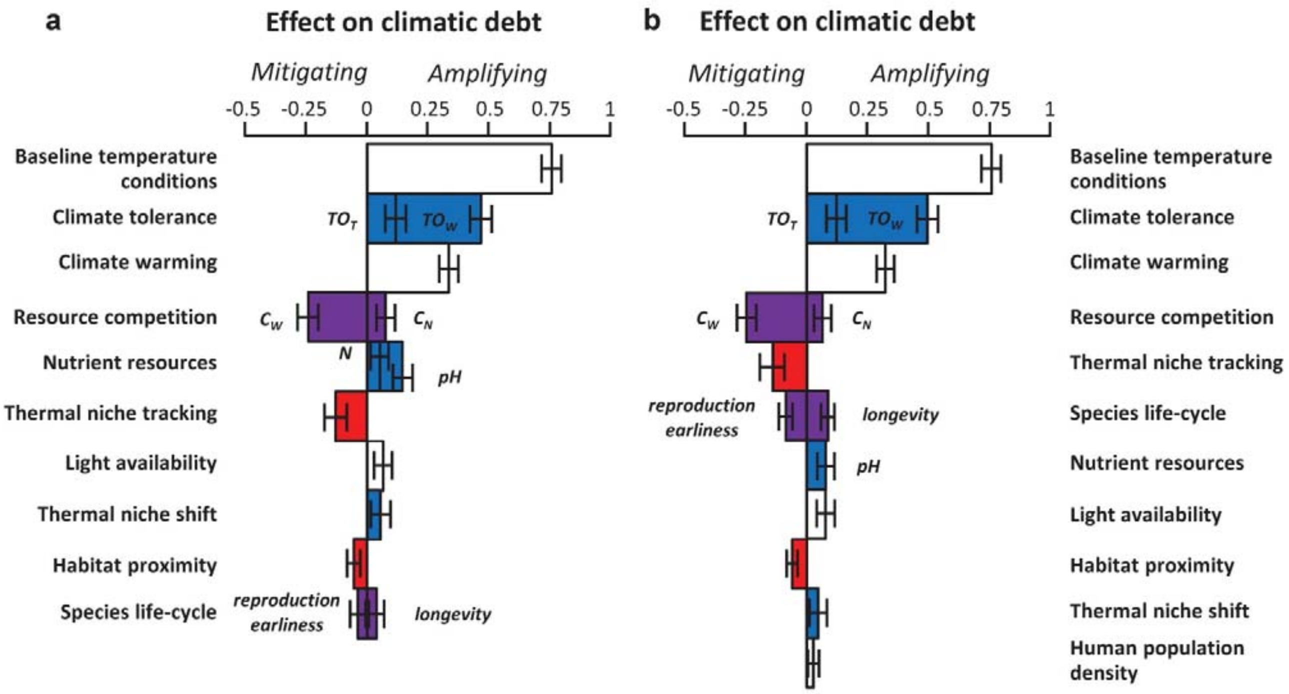

Supplementary Figure 3: Ecological determinism of the climatic debt in perturbed and non-perturbed forests. (**a**) Perturbed forests (PLS3). (**b**) Non-perturbed forests (PLS4). PLS3 and PLS4 are fitted from the same set of explanatory variables than PLS0 on two subsamples of 17,954 and 27,852 floristic observations, respectively (see Methods section in the main text). The effect of each variable is measured by the mean slope of the climatic debt *versus* this variable over the 5,000 subsamples of each model. Error bars show their 95% confidence intervals. Only significant variables are shown (bootstrap test for difference of slope values to 0: $P < 0.01$, $n = 5{,}000$), and classified by categories of factors. Temperature heterogeneity, temporal change in species habitat aggregation, road proximity, precipitation change, baseline precipitation conditions and time were not significant in both models (Supplementary Data 2). Soil nitrogen content and human population density were not significant in perturbed and non-perturbed forest plant communities, respectively. Factors involved in species' persistence and migration are specified in blue and red, respectively; purple depicts factors involved in both species' persistence and migration; white depicts environmental pressures for species. P = baseline precipitation conditions; T = baseline temperature conditions; TO_T = thermal-stress tolerance; TO_W = water-stress tolerance; C_N = interspecific competition for soil nitrogen; C_W = interspecific

competition for water; *N* = soil nitrogen content; *pH* = soil acidity.

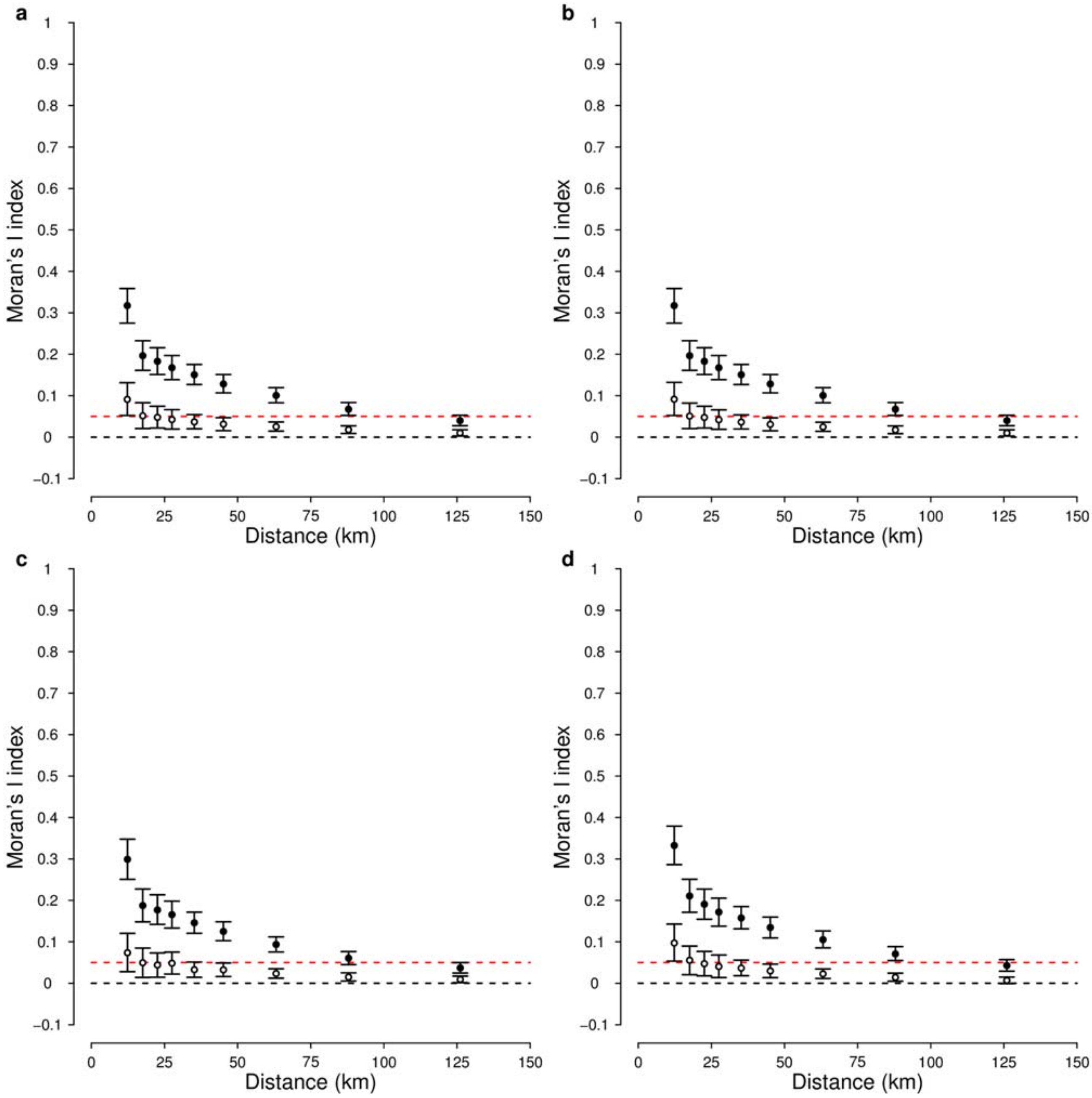

Supplementary Figure 4: Spatial correlograms computed from the climatic debt and residuals of the PLS models. (**a**) Results for the PLS1 model. (**b**) Results for the PLS2 model. (**c**) Results for the PLS3 model. (**d**) Results for the PLS4 model. Black and white dots are mean values of the Moran's *I* index computed on the climatic debt and the model residuals (n = 5,000 bootstraps), respectively. Error bars show 95% confidence intervals. The red dotted line depicts the threshold of spatial autocorrelation significance (i.e. $I = 0.05$).

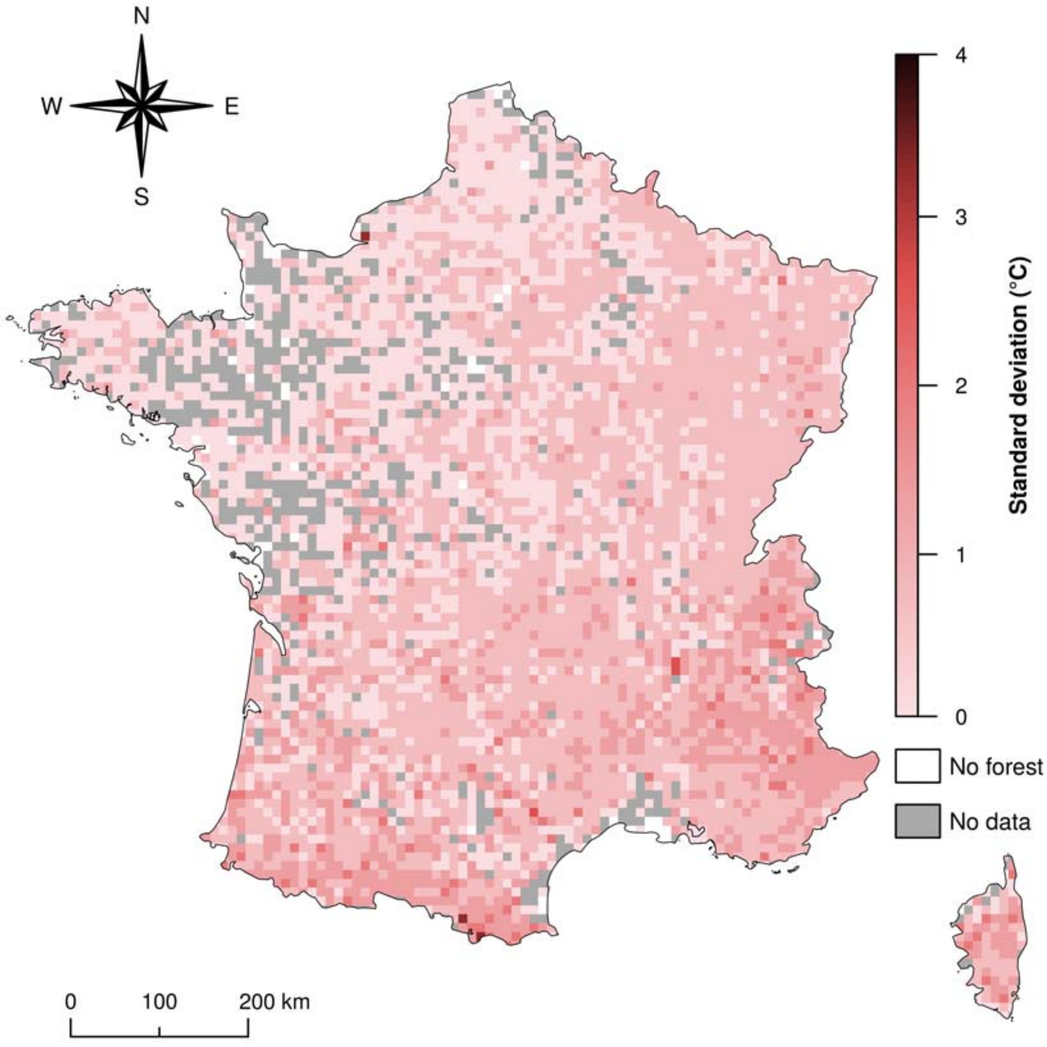

Supplementary Figure 5: Map of the local standard deviation of the observed temperature lag (*dT*) in plant communities. *dT* is computed as the difference between the temperature conditions (*CrT*) and the bioindicated temperatures (*FrT*) from the forest herbaceous plant communities**.** The map shows standard deviation values in a 10 km grid (i.e. the same grid used in Fig. 4) computed from the 5,000 subsamples used to fit the PLS0 model. The standard deviation ranges from 0 to 4°C across France and is materialized by a color gradient ranging from light red to dark red (a detailed color scale is provided to the right of the map). White and gray pixels are areas without forest territory and without

data, respectively.

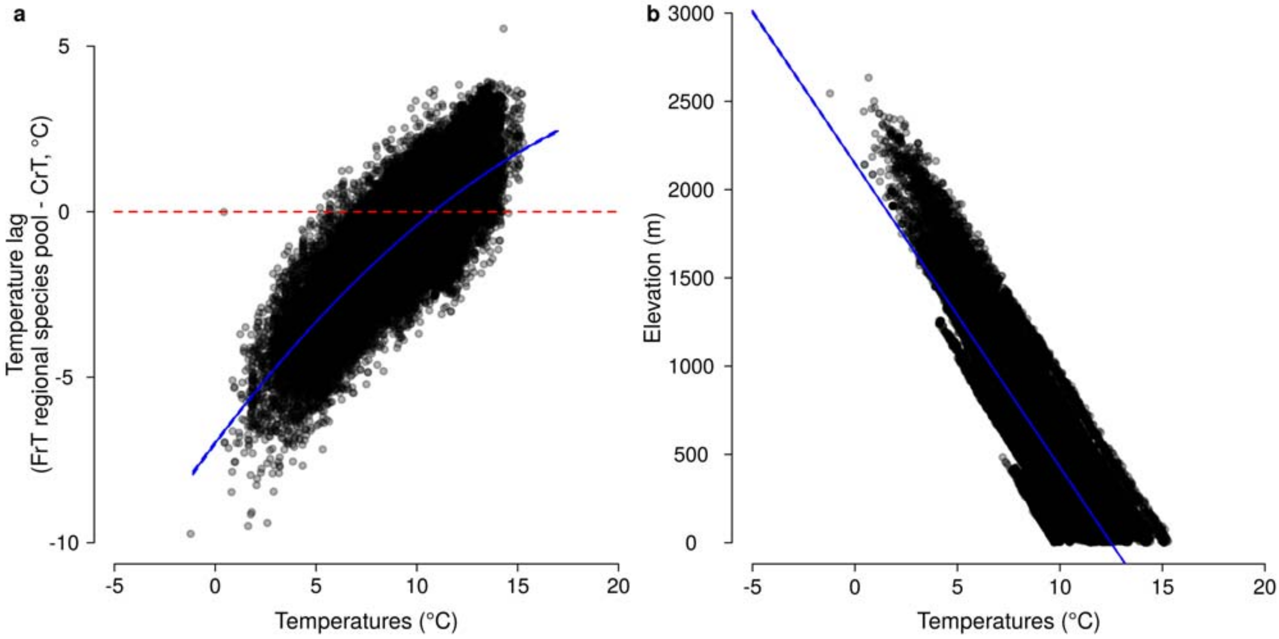

Supplementary Figure 6: Ecological explanation of the effect of temperature conditions on the climatic debt. (**a**) Insufficient recovery of the climatic debt by the regional species pool in warm conditions. For each floristic survey (*n* = 67,289), the temperature lag between maximal temperatures bioindicated from the species pool occurring within 10 km radius of the focal survey (*FrT regional species pool*) and temperatures observations (*CrT*) are computed, and its curvilinear relationship (solid blue lines: $R^2 = 0.63$; comparison to null model through *F*-test: $P < 0.00001$; comparison to linear model through *F*-test: $P < 0.00001$) with baseline temperature conditions is plotted. The climatic debt increases in warm conditions demonstrating that the regional species pool cannot totally counteract the climate warming in such conditions by providing climate-adapted species to local communities. *FrT regional species pool* is the hottest *FrT* value that we expect considering the regional species pool (i.e. the set of plant species found within a 10 km radius around the focal community and is considered as the set of species from which the focal community has been assembled in the field). It was computed for each plant community as the *FrT* value corresponding to 97.5% of the distribution of bioindicated temperatures achieved by conducting 3,000 permutations in species assemblage from the regional species pool. Every floristic survey located above the red dotted lines (i.e. temperature lag = 0) is in

climatic debt situation. (**b**) Temperatures decrease with elevation. The temperatures at the scale of the French metropolitan territory is highly dependent to the topography (solid blue lines: slope = -172, R^2 = 0.68; comparison to null model through *F*-test: $P < 0.00001$; n = 67,289).

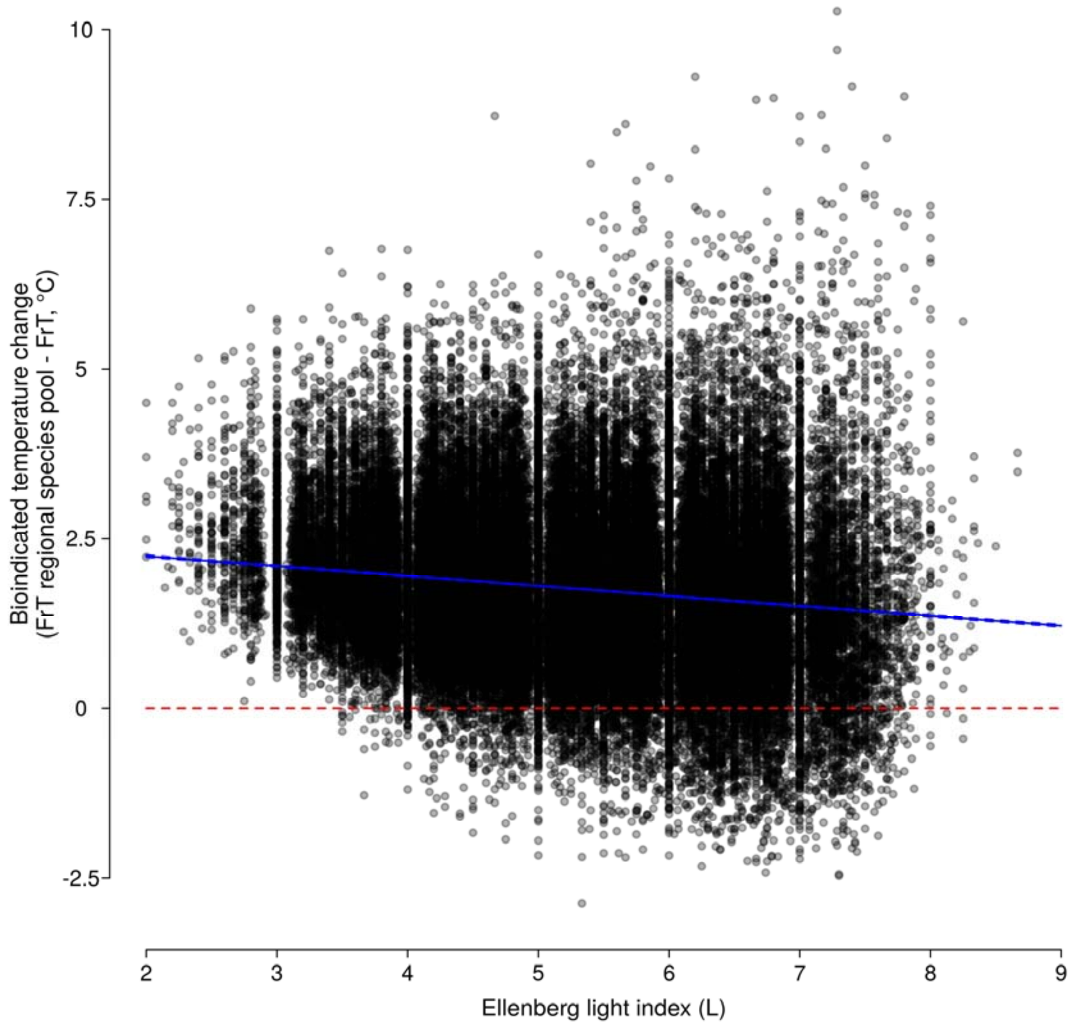

Supplementary Figure 7: Relationship between the regional bioindicated temperatures and light availability in forest stands. The figure shows a decrease of gain in warm-adapted species from the regional species pool along light availability gradient in the forest stands. The solid blue line represents the significant linear relationship (slope = -0.15, $R^2 = 0.02$; comparison to null model through *F*-test: $P < 0.00001$). For each floristic survey ($n = 67{,}289$), the bioindicated temperature difference between maximal temperatures bioindicated from the species pool occurring within 10 km radius of the focal survey (*FrT regional species pool*) and temperatures bioindicated from the floristic assemblage observed in the focal survey (*FrT*) was computed in order to assess the potential gain in warm-adapted species immigrating from the regional species pool. The Ellenberg light index is a proxy of light

availability in forest stands which increases with opening forest. Every floristic survey located above the red dotted lines (i.e. bioindicated temperature change = 0) shows a potential gain in warm-adapted species from the regional species pool which could participate to the decrease of the climatic debt in forest plant communities.

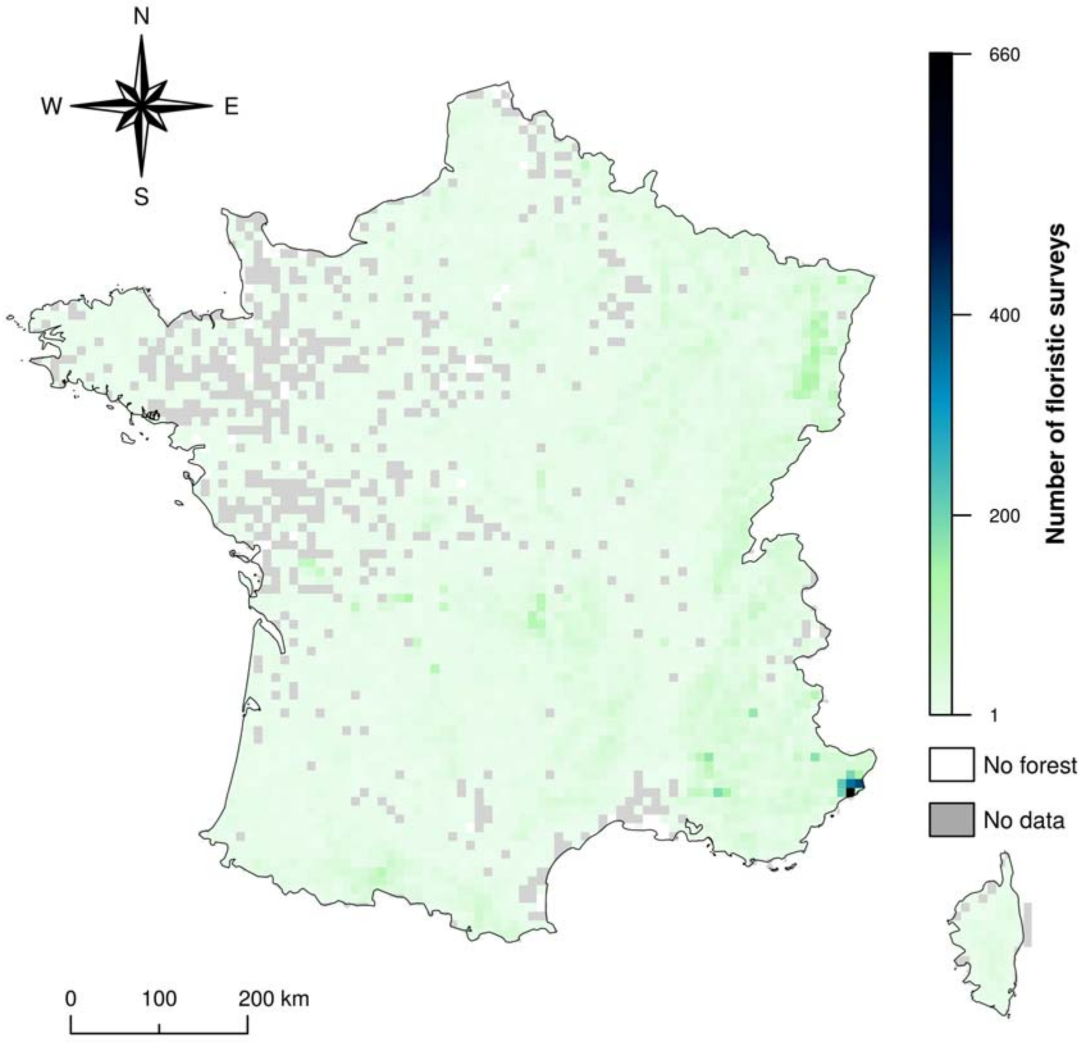

Supplementary Figure 8: Map of the density of floristic surveys available across France. On average, 12 surveys (± 20 st. dev.) were available per 100 km² area. Across France, *n* = 67,289 surveys were available in total. The number of floristic surveys per 100 km² area ranges from 1 to 660 across France and is materialized by a color gradient ranging from light green to dark blue (a detailed color scale is provided to the right of the map). White and gray pixels are areas without forest territory and without floristic survey, respectively.

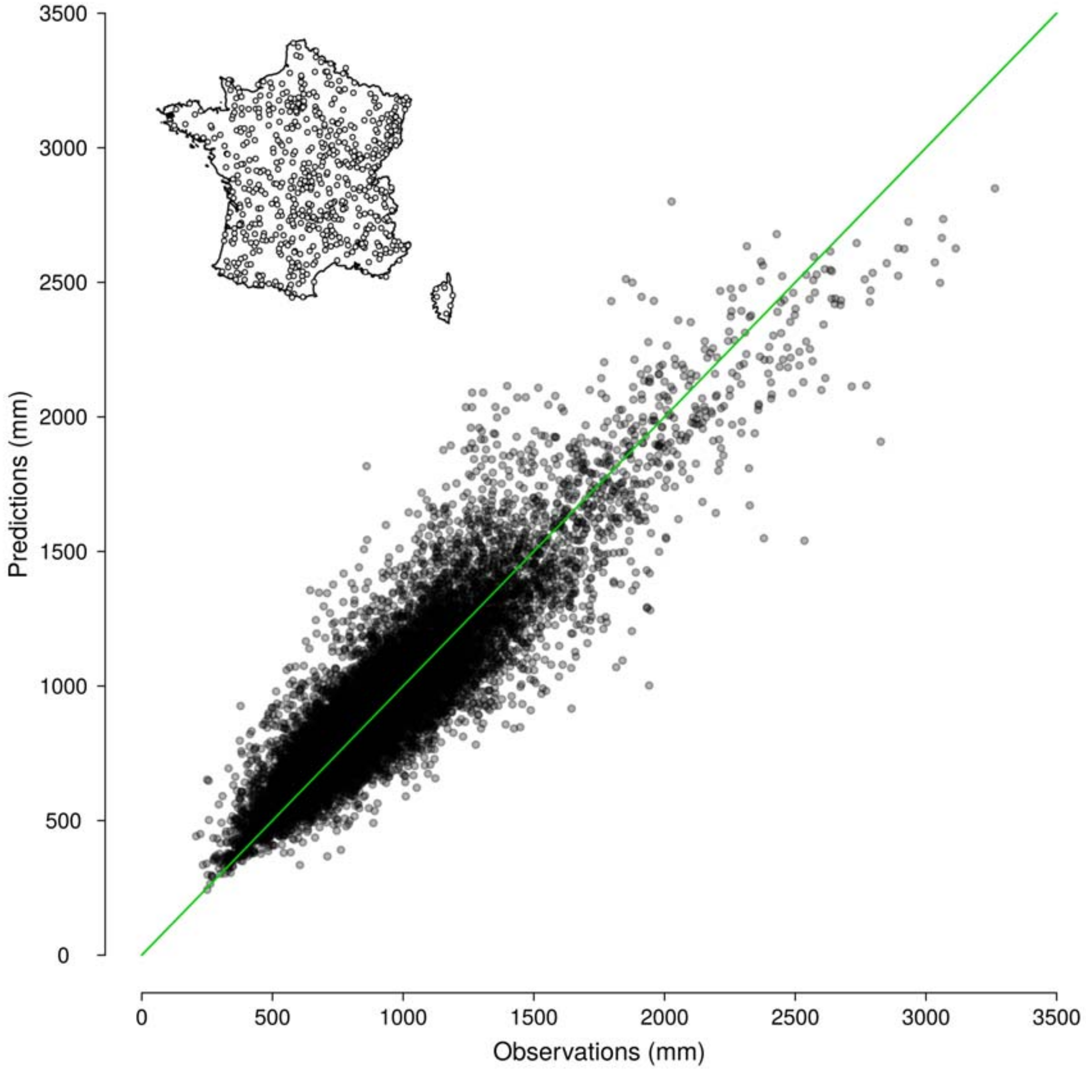

Supplementary Figure 9: Validation of the climate model predicting the annual precipitations. R^2 = 0.83 and RMSD = 132 mm for 17,865 independent precipitation observations sampled between 1965 and 2008. The spatial distribution of the 755 meteorological stations used for the validation is shown on the top left of the figure. The green line represents the perfect correspondence between observations and predictions (y = x). The gray scale which colors filled circles depicts an increasing gradient of observation number.

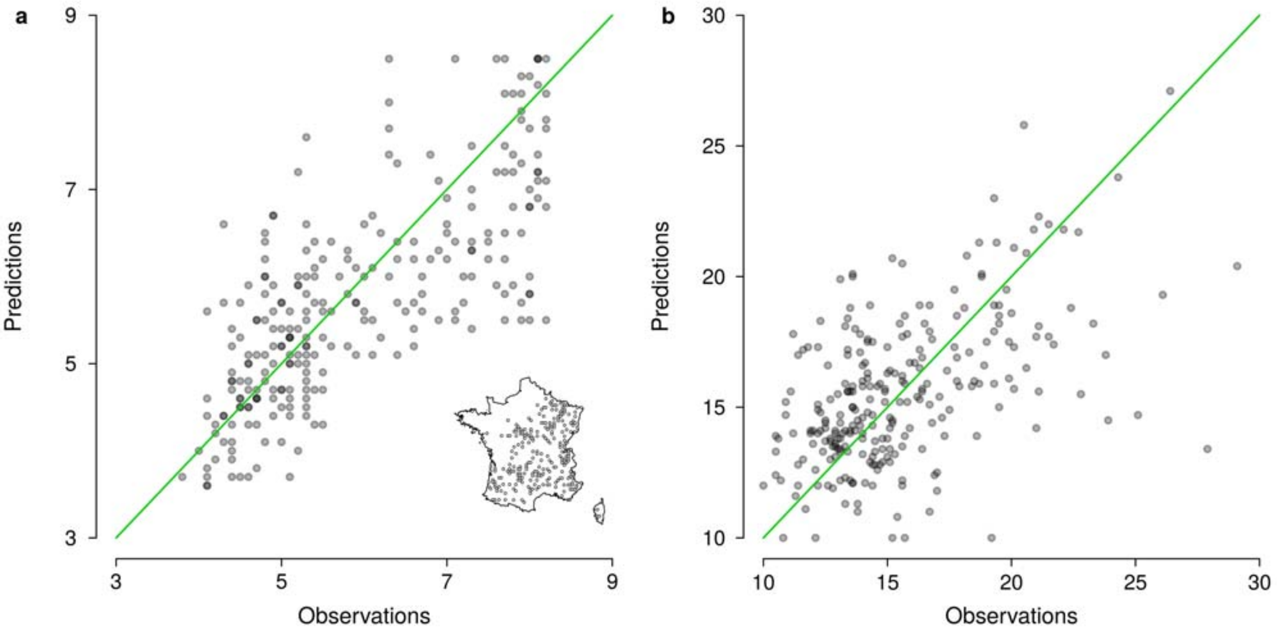

Supplementary Figure 10: Validation of the models predicting soil chemistry conditions through bioindication. (**a**) Soil acidity predictions (pH). (**b**) Soil C:N ratio predictions. Both soil pH (RMSD = 0.9) and C:N ratio (RMSD = 3.1) models are validated on the same 254 soil observations which are mapped on the bottom right of the panel (**a**). The green line represents the perfect correspondence between observations and predictions (y = x). The gray scale which colors filled circles depicts an increasing gradient of observation number.

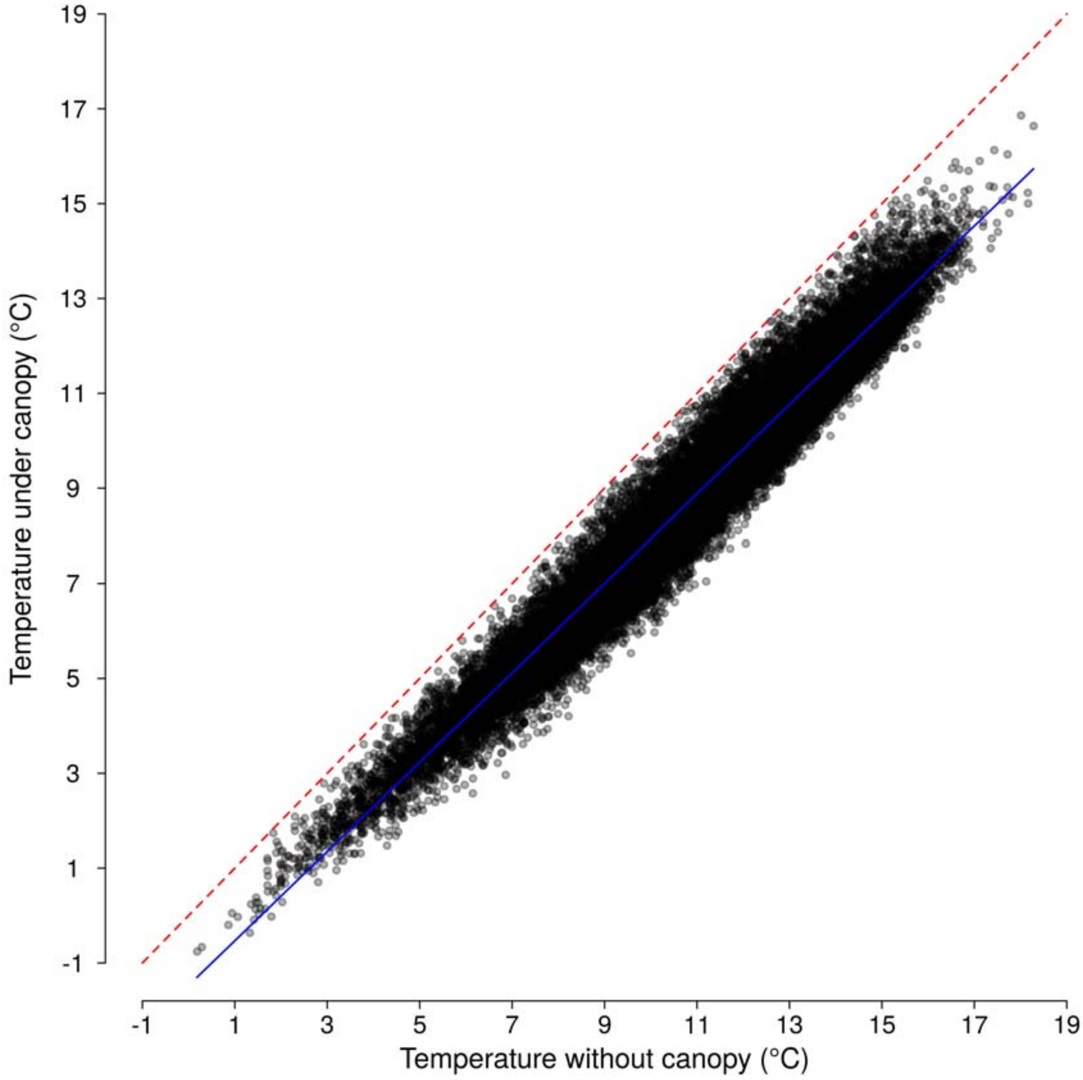

Supplementary Figure 11: Mean annual temperatures near the ground under and without forest canopy. Both temperatures were computed from the microclim model for a subset of 45,806 floristic surveys (see Methods section in the main texte and Supplementary Methods). The red dashed and solid blue lines represent the perfect correspondence (y = x) and the linear trend between both temperatures (slope = 0.941, $R^2 = 0.95$; comparison to null model through *F*-test: $P < 0.00001$), respectively.

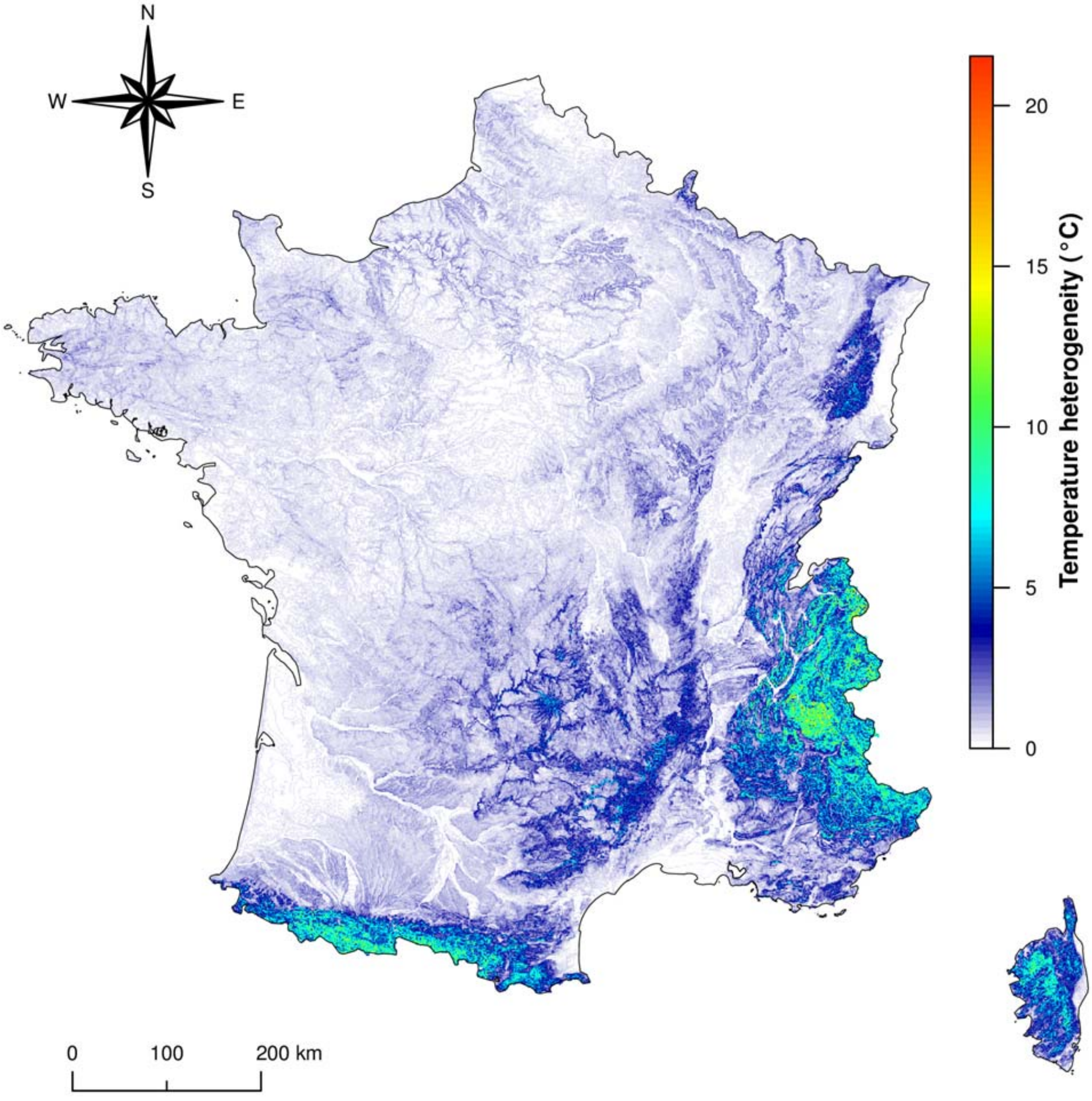

Supplementary Figure 12: Map of the temperature heterogeneity in a 1 km² pixel across France. The temperature heterogeneity is computed as the standard deviation of the 2,500 m² mean annual temperatures between 1987 and 2008 inside a 1 km² pixel. Temperature heterogeneity ranges from 0 to 22°C across France and is materialized by a color gradient ranging from white to red (a detailed color scale is provided to the right of the map).

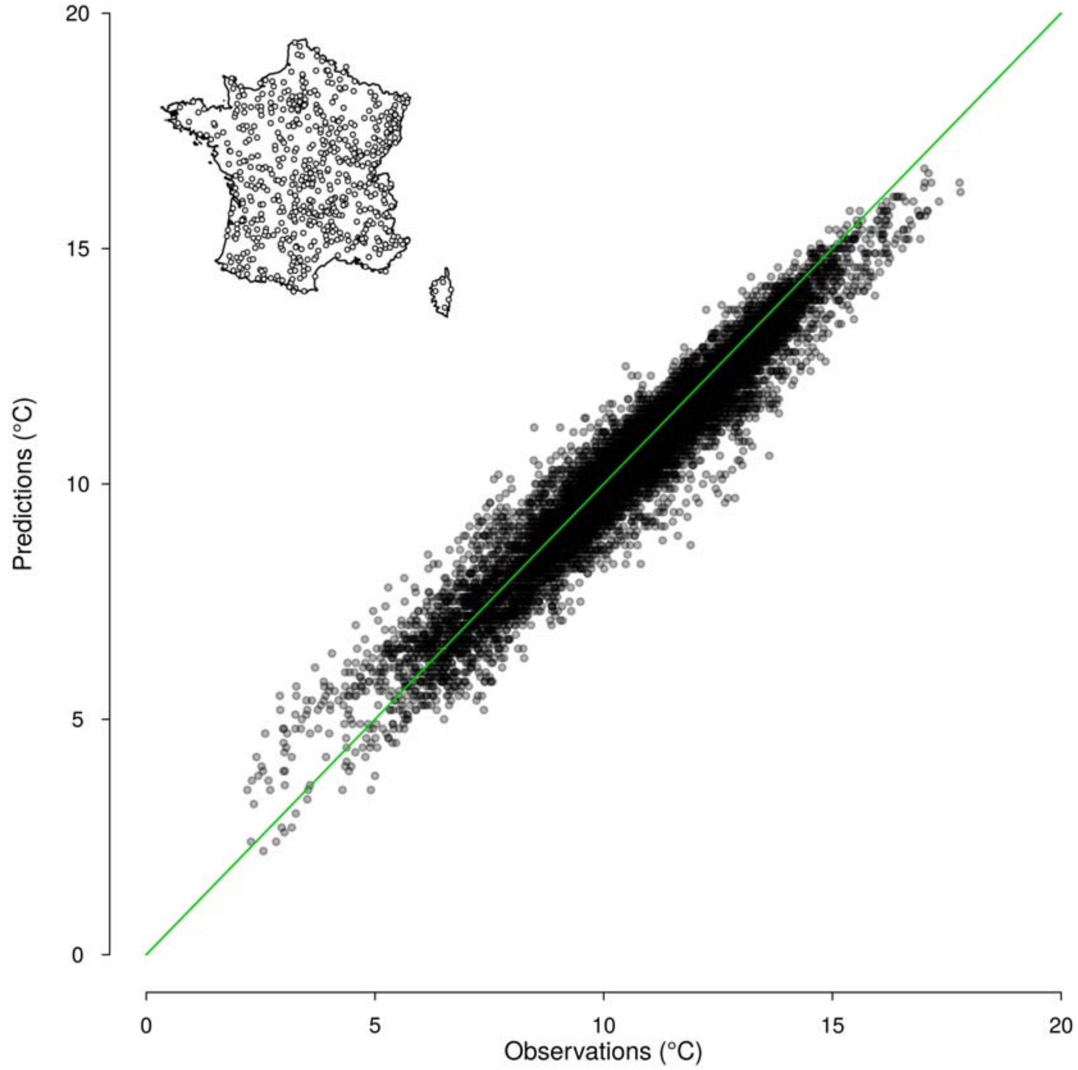

Supplementary Figure 13: Validation of the high spatial resolution temperature model. This model predicts the annual mean temperature at 2,500 m² spatial resolution with high confidence (R^2 = 0.93 and RMSD = 0.58°C for 13,620 independent temperature observations sampled between 1965 and 2008). The spatial distribution of the 602 meteorological stations used for the validation is shown on the top left of the figure. The green line represents the perfect correspondence between observations and predictions (y = x). The gray scale which colors filled circles depicts an increasing gradient of observation number.

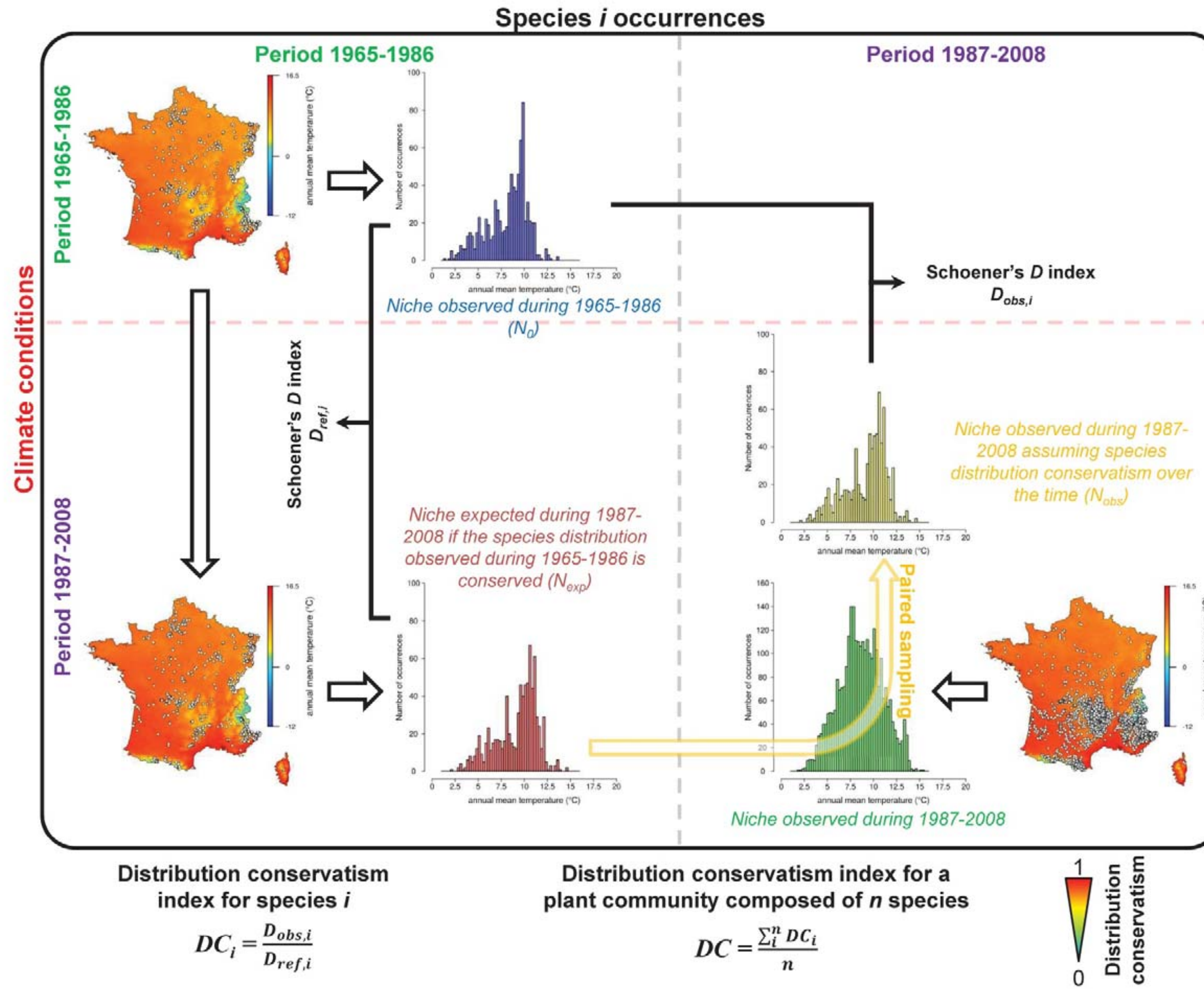

Supplementary Figure 14: Step by step description of the computation of the distribution conservatism index (*DC*).

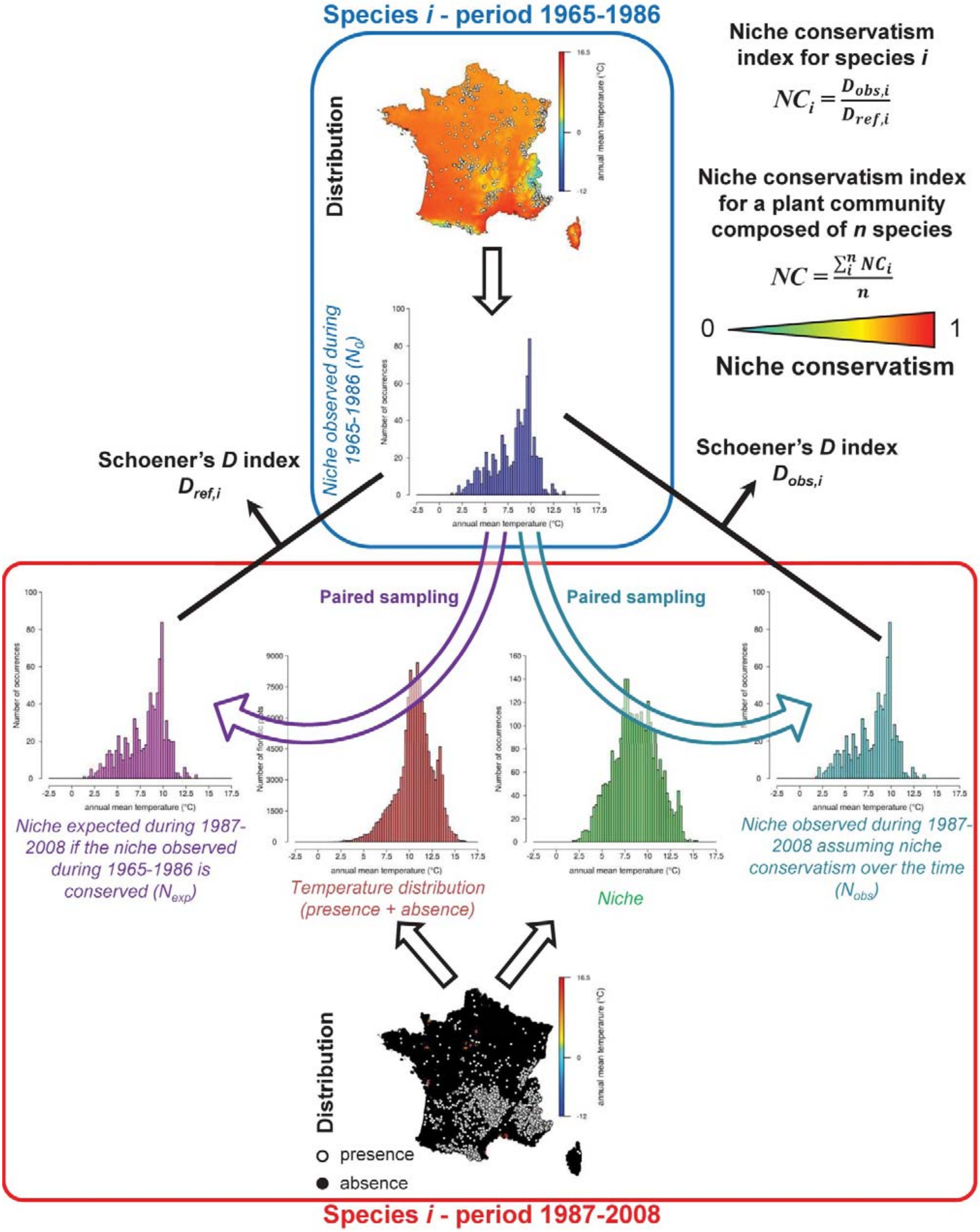

Supplementary Figure 15: Step by step description of the computation of the niche conservatism index (*NC*).

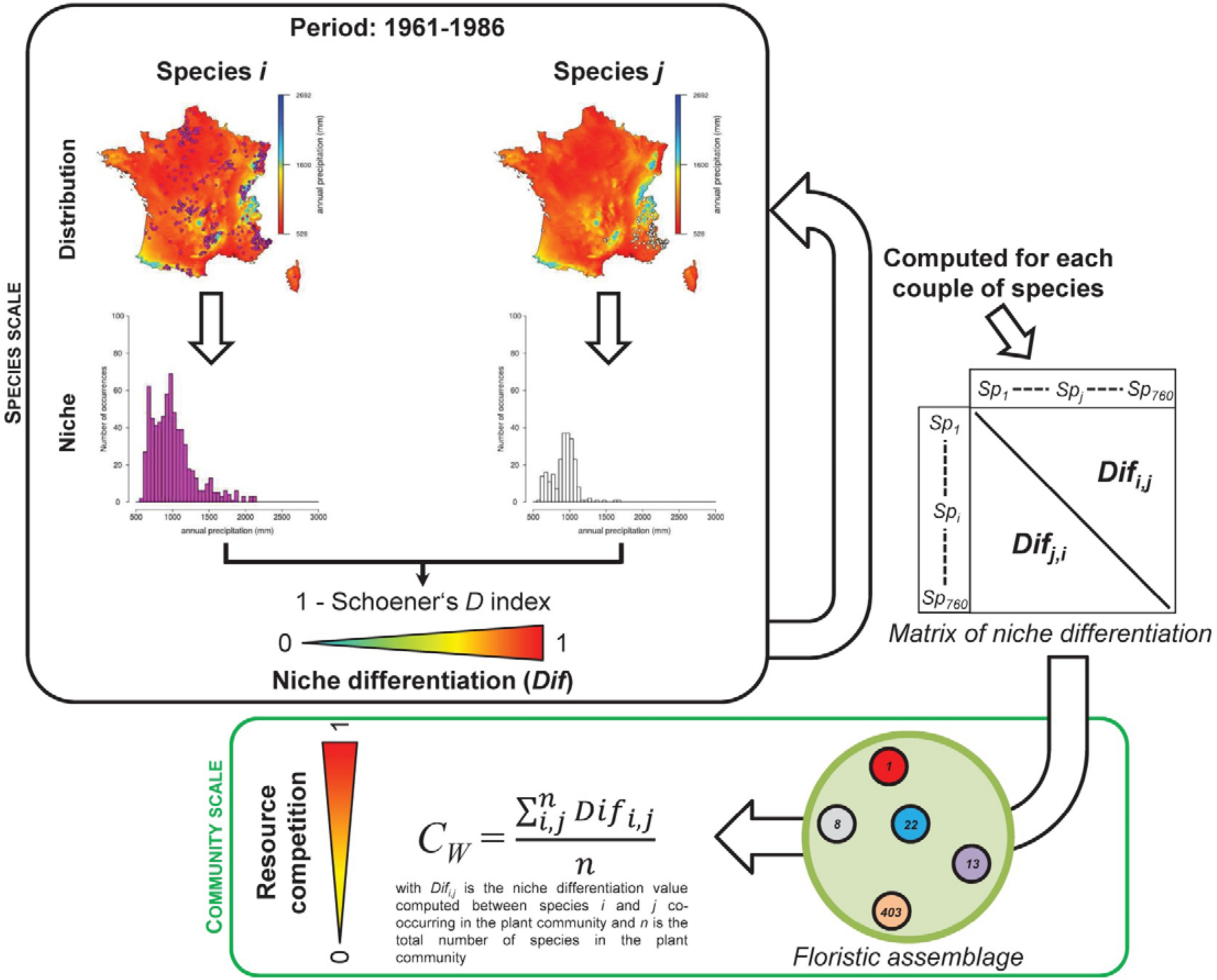

Supplementary Figure 16: Step by step description of the computation of the resource competition indices (C_W and C_N). Only the case of the interspecific competition for water resource (C_W) is shown.

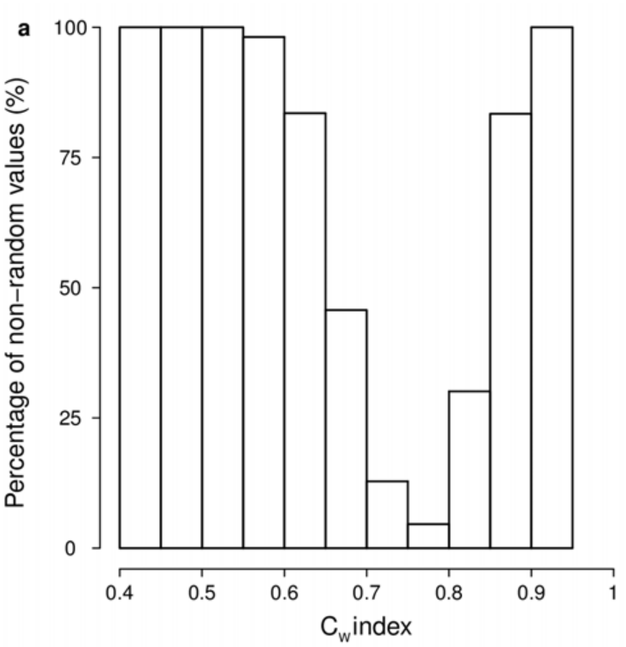

Supplementary Figure 17: Non-random species assemblages determine extreme values of resource competition indices. (**a**) Interspecific resource competition for water (C_W). (**b**) Interspecific resource competition for nitrogen (C_N).

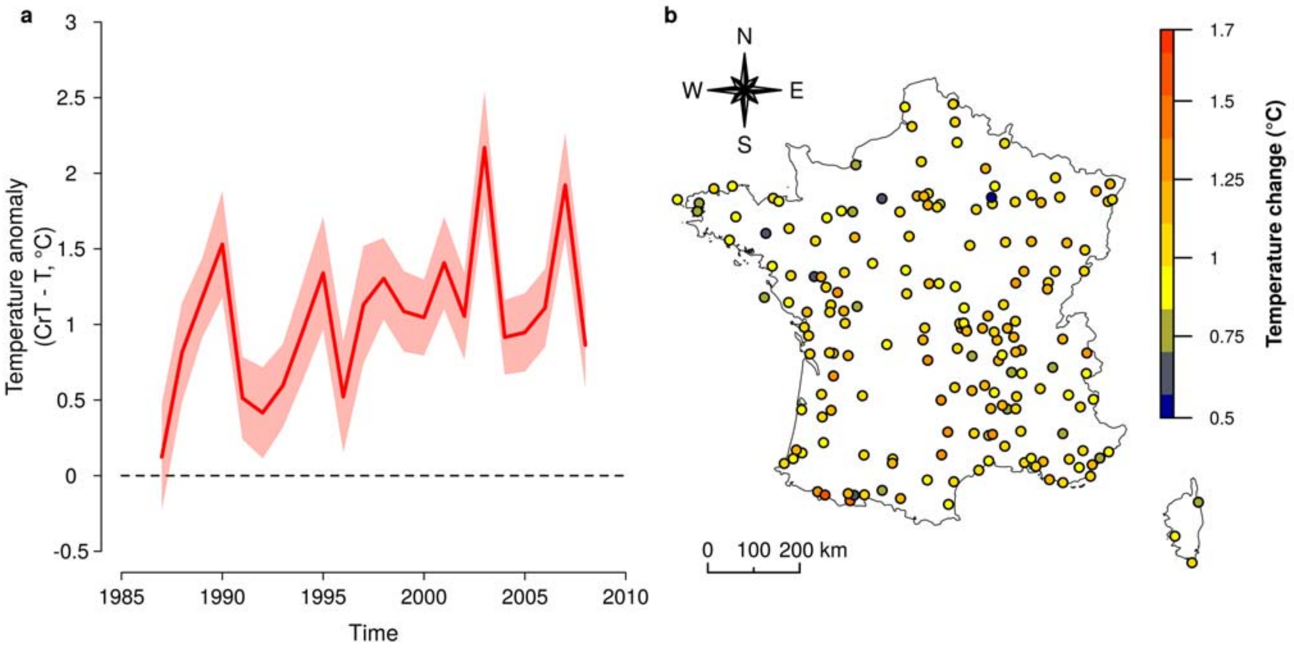

Supplementary Figure 18: Temperature changes in time and space. (**a**) Temperature anomalies trend between 1987 and 2008 (i.e. the time scale of the study) in France. Temperature anomalies are computed as the difference between yearly temperatures (*CrT*) and baseline temperature conditions over the period 1965-1986 (*T*) from 180 meteorological stations (mapped in panel (**b**)). The red line represents the mean trend and the surface represents the standard deviation. (**b**) Spatial variation of the contemporary climate warming in France. Temperature change is computed as the difference between the temperature averages over the periods 1987-2008 and 1965-1986. Temperature change ranges from 0.5 to 1.7°C across France and is materialized by a color gradient ranging from dark blue to red (a detailed color scale is provided to the right of the map).

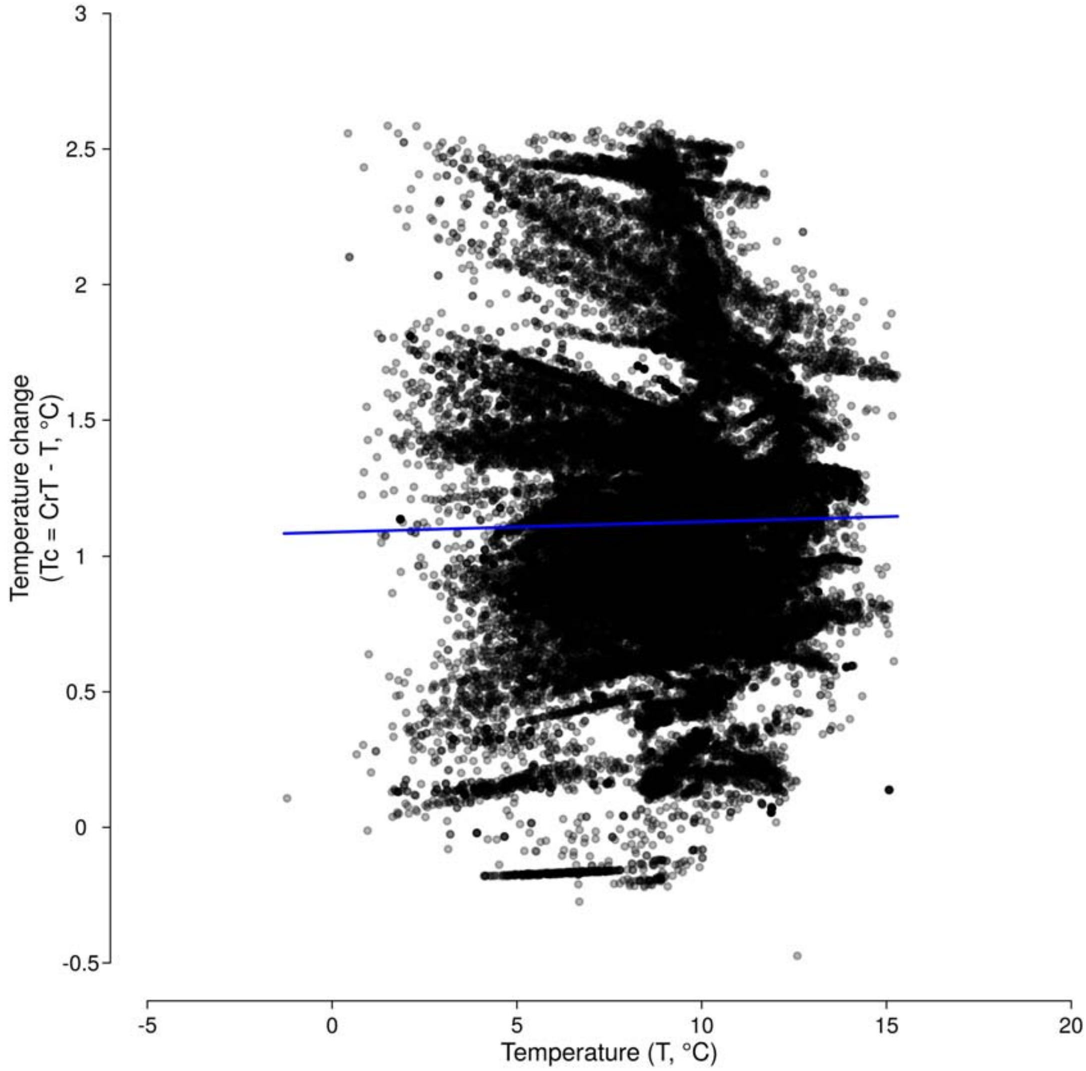

Supplementary Figure 19: Temperature change between 1965 and 2008 (*TC*) is independent to baseline temperature conditions (*T*). The blue line represents the linear trend (slope = 0.0038, R^2 = 0.0003, n = 67,289 observations).

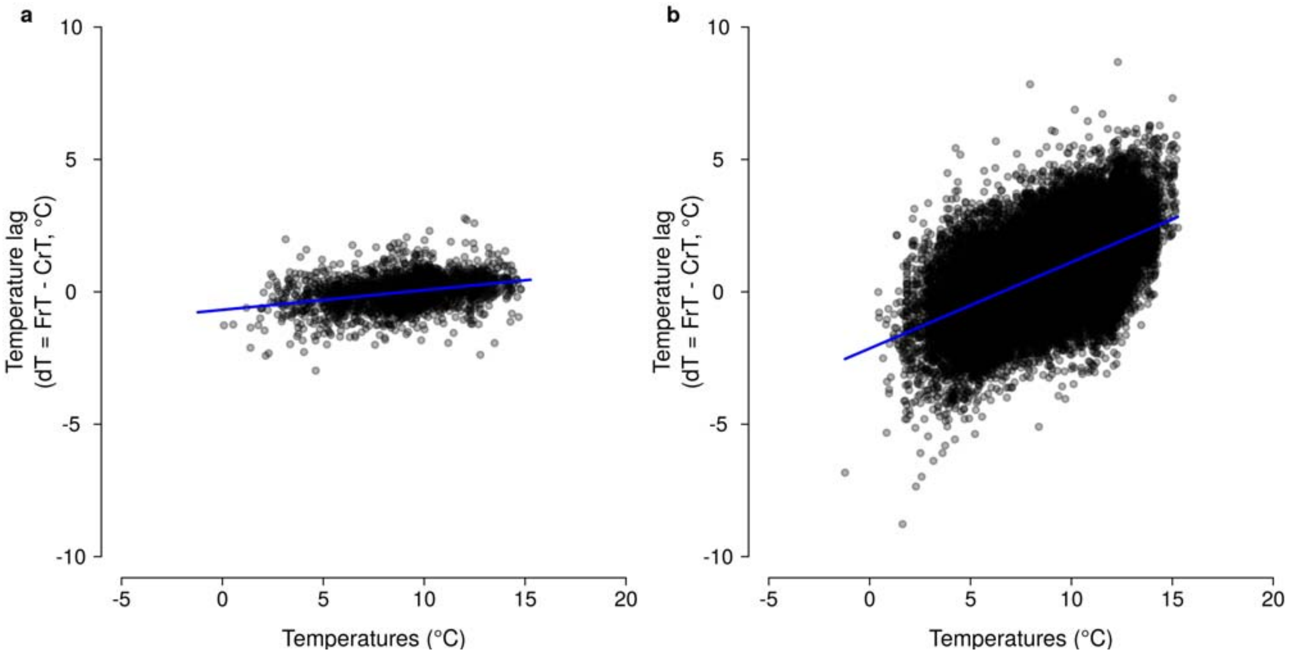

Supplementary Figure 20: Temporal change in the relationship between climatic debt (*dT*) and temperature conditions (*T*). (**a**) The relationship for the period 1975-1985 (i.e. before the contemporary climate change and over the period of the model calibration) (slope = 0.075, R^2 = 0.102, *n* = 2,987 plots). (**b**) The relationship for the period 1987-2008 (slope = 0.326, R^2 = 0.3, *n* = 67,289). The blue lines represent the linear trends.

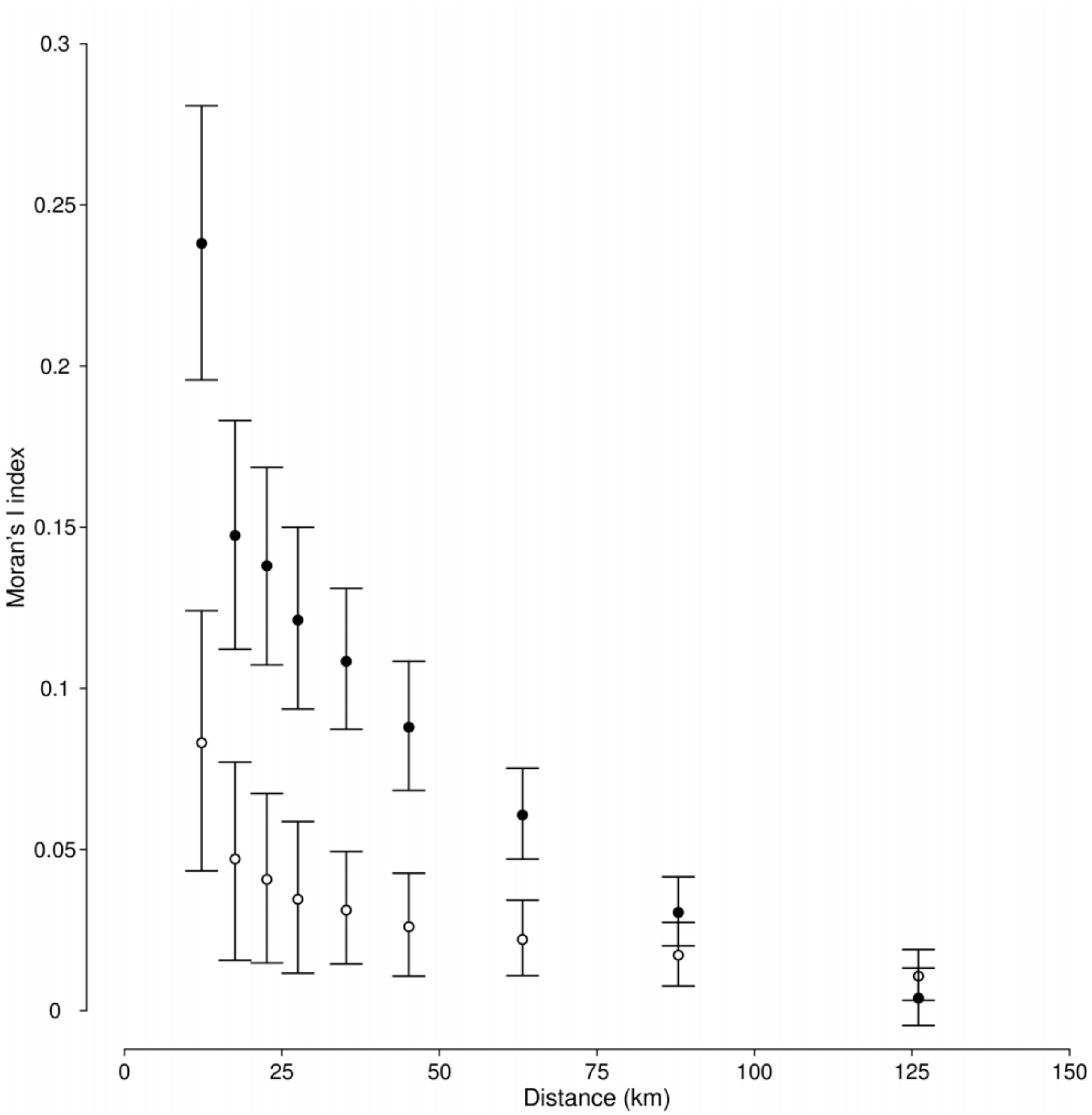

Supplementary Figure 21: Impact of baseline temperature conditions on the spatial autocorrelation. The figure shows spatial correlograms computed on residuals of PLS0 models accounting (white dots) and not accounting (black dots) for the effect of baseline temperature conditions (*T*) on the climatic debt. Mean values (dots) and 95% confidence intervals (error bars) are shown.

Supplementary Tables

Supplementary Table 1: Statistics of the parameters fitted in the PLS0 model.

Factor	Code	R^2		Slope value		
		Average	$CI_{95\%}$	Average	$CI_{95\%}$	p-value
Baseline temperature conditions	*T*	0.148	[0.13; 0.167]	0.707	[0.659; 0.753]	**0**
Contemporary temperature change	*TC*	0.094	[0.077; 0.11]	0.386	[0.35; 0.421]	**0**
Baseline precipitation conditions	*P*	0.047	[0.038; 0.056]	-0.010	[-0.054; 0.034]	0.328
Contemporary precipitation change	*PC*	0.002	[0.001; 0.004]	-0.002	[-0.033; 0.031]	0.461
Road proximity	*RP*	0.002	[0; 0.004]	-0.021	[-0.042; 0.002]	0.036
Human population density	*HPD*	0.007	[0.004; 0.009]	0.012	[-0.009; 0.032]	0.127
Temperature heterogeneity	*THET*	0.019	[0.013; 0.026]	0.019	[-0.022; 0.06]	0.172
Light availability in forest stand	*L*	0.014	[0.011; 0.017]	0.084	[0.038; 0.131]	**0**
Soil pH	*pH*	0.009	[0.006; 0.013]	0.078	[0.029; 0.124]	**0.001**
Soil nitrogen content	*N*	0.009	[0.004; 0.015]	0.061	[0.012; 0.112]	**0.007**
Thermal-stress tolerance	TO_T	0.018	[0.014; 0.024]	0.075	[0.023; 0.127]	**0.002**
Water-stress tolerance	TO_W	0.037	[0.031; 0.045]	0.378	[0.326; 0.428]	**0**
Thermal niche shift	*DC*	0.011	[0.008; 0.014]	0.068	[0.018; 0.119]	**0.003**
Species competition for water	C_W	0.014	[0.01; 0.018]	-0.218	[-0.266; -0.167]	**0**
Species competition for N-nutrition	C_N	0.012	[0.009; 0.015]	0.070	[0.027; 0.111]	**0.002**
Species longevity	*LG*	0.006	[0.004; 0.009]	0.061	[0.025; 0.096]	**0**
Thermal niche tracking	*NC*	0.019	[0.014; 0.024]	-0.173	[-0.235; -0.11]	**0**
Past species habitat patches' proximity	*HP*	0.024	[0.018; 0.031]	-0.060	[-0.085; -0.035]	**0**
Temporal changes in species habitat aggregation	*dHA*	0.032	[0.024; 0.04]	-0.034	[-0.068; 0.001]	0.028
Time	*TIME*	0.040	[0.033; 0.048]	-0.029	[-0.064; 0.004]	0.046

Average values and 95% confidence intervals ($CI_{95\%}$) of R^2 and slope values were computed from the 5,000 bootstrapped PLS0 models. The p-values show the results of the bootstrap test for difference of slope values to 0. The significant factors are specified by bold p-values (i.e. p-value < 0.01).

Supplementary Table 2: Test of the difference between the effects estimated from the PLS0 model and PLS1 to PLS4 models.

Factor	Code	PLS1	PLS2	PLS3	PLS4
Baseline temperature conditions	*T*	0.444	0.279	0.313	0.293
Contemporary temperature change	*TC*	0.035	0.038	0.142	0.027
Baseline precipitation conditions	*P*	0.987	0.967	0.847	0.962
Contemporary precipitation change	*PC*	0.93	0.866	0.765	0.912
Road proximity	*RP*	0.763	0.793	0.901	0.559
Human population density	*HPD*	0.594	0.628	0.703	0.559
Temperature heterogeneity	*THET*	0.878	0.901	0.753	0.872
Light availability in forest stand	*L*	0.935	0.865	0.844	0.95
Soil pH	*pH*	0.97	0.973	0.897	0.984
Soil nitrogen content	*N*	0.953	0.94	0.965	0.874
Thermal-stress tolerance	TO_T	0.616	0.675	0.53	0.449
Water-stress tolerance	TO_W	0.949	0.968	0.727	0.949
Thermal niche shift	*DC*	0.934	0.962	0.921	0.884
Species competition for water	C_W	0.851	0.792	0.797	0.687
Species competition for N-nutrition	C_N	0.956	0.968	0.94	0.952
Species longevity	*LG*	0.959	0.931	0.688	0.569
Thermal niche tracking	*NC*	0.796	0.748	0.657	0.779
Past species habitat patches' proximity	*HP*	0.876	0.754	0.863	0.96
Temporal changes in species habitat aggregation	*dHA*	0.818	0.861	0.284	0.769
Time	*TIME*	0.688	0.747	0.91	0.597

No significant difference was observed whatever the variable considered (significance threshold $\alpha = 0.01$). PLS1, PLS2, PLS3 and PLS4 columns show the p-value of the bootstrap test ($n = 5{,}000$) comparing each effect (i.e. slope values) estimated from the PLS0 models to PLS1, PLS2, PLS3 and PLS4 models, respectively.

Supplementary Table 3: Uncertainty in explanatory variables and its effect on parameter estimations.

variable	*n*	uncertainty (variance ratio)	PLS0's slope (*b**)	corrected slope (*b*)	*b*-*b**
T	180	0.967	0.707	0.731	0.024
TC	3,960	1.05	0.389	0.367	-0.022
P	429	1.073	-0.01	-0.009	0.001
PC	9,438	1.047	-0.002	-0.001	0.001
pH	254	1.115	0.078	0.070	-0.008
N	254	1.432	0.061	0.043	-0.018

The uncertainty in variable computation is assessed by the ratio between variances in true observations and estimation[1]. Regression dilution impacts a variable when the ratio value is less than 0, which leads to underestimate parameters in regression. The impact of uncertainty on parameter estimation can be assessed through the comparison of original regression parameter fitted in the PLS0 model (*b**) and its correction (*b*) accounting for uncertainty (*b** = *b* x *uncertainty*[1]). *n* is the number of observations used to compute the uncertainty. The definition of variables is available in Table 1 and in Supplementary Data 1.

Supplementary Table 4: Test of the difference between the effects estimated from the PLS3 and PLS4 models.

Factor	Code	p-value
Baseline temperature conditions	*T*	0.939
Contemporary temperature change	*TC*	0.791
Baseline precipitation conditions	*P*	0.524
Contemporary precipitation change	*PC*	0.830
Road proximity	*RP*	0.657
Human population density	*HPD*	0.934
Temperature heterogeneity	*THET*	0.888
Light availability in forest stand	*L*	0.843
Soil pH	*pH*	0.800
Soil nitrogen content	*N*	0.820
Thermal-stress tolerance	TO_T	0.920
Water-stress tolerance	TO_W	0.729
Thermal niche shift	*DC*	0.868
Species competition for water	C_W	0.875
Species competition for N-nutrition	C_N	0.841
Species longevity	*LG*	0.062
Thermal niche tracking	*NC*	0.905
Past species habitat patches' proximity	*HP*	0.847
Temporal changes in species habitat aggregation	*dHA*	0.606
Time	*TIME*	0.621

No significant difference was observed whatever the variable considered (bootstrap test for difference in slopes values between PLS3 and PLS4 models, significance threshold $\alpha = 0.01$, $n = 5{,}000$).

Supplementary Table 5: Data used to run the microclim model.

Variable category	Parameter	Type of data	Original resolution		Final resolution		Source
			Spatial	Time	Spatial	Time	
climate conditions	mean air temperature	model	1 km2	monthly from 1987 to 2008			*[2,3]*
	minimum air temperature	model	0.5° (~2120 km²)	monthly from 1987 to 2008	1 km²		CRU TS v. 3.23 *[4]*
	maximum air temperature	model	0.5° (~2120 km²)	monthly from 1987 to 2008	1 km²		CRU TS v. 3.23 *[4]*
	rainfall	model	1 km²	monthly from 1987 to 2008			*[2,3]*
	rainy days	model	10' (~2004 km²)	monthly average for 1961-1990 period	1 km²		CRU CL v. 2.0 *[5]*
	relative humidity	model	10' (~2004 km²)	monthly average for 1961-1990 period	1 km²		CRU CL v. 2.0 *[5]*
	cloud cover	model	0.5° (~2120 km²)	monthly from 1987 to 2008	1 km²		CRU TS v. 3.23 *[4]*
soil conditions	soil moisture	model	1 km²	monthly from 1987 to 2008			*[2,3,6]*
	soil class	model	1 km²	average conditions over the past 50 years			SoilGrids1km *[7]*
	soil clay content	model	1 km²	average conditions over the past 50 years			SoilGrids1km *[7]*
	soil bulk density	model	1 km²	average conditions over the past 50 years			SoilGrids1km *[7]*
topography	altitude	model	1 km²	computed between 1987 and 2001			BD ALTI® (http://professionnels.ign.fr/bdalti)
	slope	model	1 km²	computed between 1987 and 2001			BD ALTI® (http://professionnels.ign.fr/bdalti)
	aspect	model	1 km²	computed between 1987 and 2001			BD ALTI® (http://professionnels.ign.fr/bdalti)
	hillshade	model	1 km²	computed between 1987 and 2001			computed from BD ALTI®
forest description	shading canopy	observation	625 m²	date of the floristic observation			IGN-IFN database *[8]*
	canopy height	observation	225 m²	date of the floristic observation			IGN-IFN database *[8]*

Only the variables which differ from the default parametrization of the microclim model are listed in the Table. Original and final resolutions define spatial and temporal resolutions of the data provided by the source and used to parametrize the model, respectively. Only the final

spatial and/or temporal resolutions are mentioned when a variable is transformed. References of data are given in brackets in the source field.

Supplementary Table 6: List of exotic tree species considered.

Species	*n*
Abies grandis	273
Abies nordmanniana	84
Acer negundo	33
Aesculus hippocastanum	136
Ailanthus altissima	13
Cedrus atlantica	419
Cupressus sempervirens	28
Juglans regia	1,068
Larix kaempferi	140
Liriodendron tulipifera	3
Picea sitchensis	258
Pinus nigra	1,915
Pinus nigra subsp. nigra	478
Pinus strobus	78
Populus robusta	0
Populus trichocarpa	1
Populus x-canadensis	208
Pseudotsuga menziesii	2,285
Quercus cerris	35
Quercus palustris	8
Quercus rubra	422
Robinia pseudoacacia	2,422
Sorbus x-intermedia	0
Tsuga heterophylla	6

n = species occurrence in the database.

Supplementary Notes

Supplementary Note 1: Baseline temperature conditions matter to explain the climatic debt.

Circularity issues are observed in a model when one or more explanatory variables (X_i) are involved in the computation of the dependent variable (*Y*), or when similar data are used to compute both the X_i variable(s) and the *Y* variable. In the present study, this situation does not occur between the climatic debt (*dT*) and the set of explanatory variables investigated, notably the baseline temperature conditions (i.e. annual mean temperature during 1965-1986, *T*). Indeed, the climatic debt ($dT = CrT – FrT$) is not computed from *T* but from the difference between the yearly interpolated temperature (*CrT*) and the floristically reconstructed temperature (*FrT*) over a completely different time period (1987-2008).

Despite *CrT* is being related to *T* due to high geographic and topographic constraints on temperature conditions[3], both variables differ in time due to the yearly anomalies that exist between yearly temperature conditions over 1987-2008 and baseline temperature conditions averaged over 1965-1986 (Supplementary Fig. 18a). Both variables also differ in space because climate warming between 1965-1986 and 1987-2008 is not uniform across the French territory (Supplementary Fig. 18b). These spatio-temporal differences between *T* and *CrT* limit any circularity issue between *dT* and *T*.

Circularity between *Y*- and X_i-variables is a modelling issue if it leads to a strong relationship between these two set of variables. However, circularity does not necessarily imply any relationship between variables. For instance, we observed no relationship between climate warming (*TC*) and baseline temperature conditions (*T*) ($R^2 = 0.0003$; Supplementary Fig. 19) while both variables can be considered as circular ($TC = CrT – T$). Indeed, climate has warmed independently to baseline climate conditions in France. It means that even in a case of circularity observed between variables, circularity is not necessarily an issue if it does not lead to high correlation between variables.

Moreover, we consider that the relationship found between *dT* and *T* during the studied period (1987-2008) has an ecological meaning. Under the assumption that forest plant communities are

reshuffled as fast as the temperature rises, then we should not expect any change, over time, in the relationship between *dT* and *T*. Here, however, we observed that the slope of the relationship increases from 0.075 during the 1975-1985 period to 0.326 during the 1987-2008 period (comparison of slope values through Student's *t*-test: $P < 0.0001$; Supplementary Fig. 20). This result demonstrates that the response of forest plant communities to the contemporary climate change varies across the French territory in accordance to the baseline temperature conditions. Warmer is the baseline climate conditions higher is the climatic debt, likely due to a deficit of warm-adapted species within the regional species pool (see the Results section in the main text for more details). Moreover, such an ecological pattern confirms previous results which demonstrated that lowland forest plant communities (observed in warmer conditions than highland forest plant communities) are lagging more behind climate change than highland forest plant communities[2].

Finally, we included baseline temperature conditions as an explanatory variable in our modelling framework because it captures a large amount of the spatial autocorrelation signal observed in the climatic debt. Indeed, we observed that a positive spatial autocorrelation signal remains in the first distance classes of the model residuals when baseline temperature conditions are not accounted for (Supplementary Fig. 21). Not considering this variable in models could have strong consequences on the model outputs, notably by incorporating a bias and inverting the relationships fitted by the PLS regression[9,10].

All these arguments justify the use of the baseline temperature conditions as an important explanatory variable to explain the climatic debt.

Supplementary Methods

Modeling approach to infer temperature from the floristic assemblage.

Our modeling approach is based on a transfer function that relates species composition within floristic assemblages to temperatures. This approach is similar to transfer functions commonly used in paleoecological studies which relate pollen (for instance) assemblages to temperatures in order to reconstruct past climatic conditions[11-13]. Our transfer function combined weighted averaging partial least squares[14] (WA-PLS) and Breiman's random forest[15] (BRF) regressions to predict temperatures from the species assemblage of each floristic survey at a given location and year (hereafter, floristically reconstructed temperatures, *FrT*). Using BRF regression techniques in combination with WA-PLS allows the non-linearity in species temperature relationships and species dependences to be accounted for.

A training data set was used to calibrate the model for inferring *FrT*. The 2,987 floristic surveys included in the training data set were sampled during 1975-1985 (i.e. before the warm period of 1987–2008) in order (i) to avoid bias due to plant responses to climate change in the model and (ii) to fit the model on a relatively stable climatic period assuming an equilibrium state between plant and temperature conditions. This assumption was verified by the absence of climatic debt observed in the French forest plant communities between 1965 and 1986[2]. The floristic surveys were selected to be 500 m apart at least (to minimize spatial autocorrelation issues during model calibration). To ensure a good model fit, and to avoid misidentification issues of rare species, we focused on the most common herbaceous species (i.e. frequency ≥ 5 occurrences) and floristic surveys that were composed of at least 5 of these species. To further avoid misidentification issues, we mostly focused on the species level (except for 25 common and non-problematic sub-species; Supplementary Data Set 3). A total of 760 herbaceous species from 61 phylogenetic families were selected for model calibration (Supplementary Data 3). To avoid overprediction from our model, the set of 67,289 floristic surveys used to investigate the climatic debt in the present study followed the same sampling criterion as the training data set.

Using the training data set, we first calibrated a WA-PLS model that linked the floristic assemblage (*floAss*; i.e. the *X*-variables) of each of the 2,987 floristic surveys with the corresponding temperatures (*CrT*; i.e. the *Y*-variable) predicted from a climate model (see the Methods section in the main document) at the year and location of the floristic inventory: $CrT = f_{(floAss)}$ (model 1). WA-PLS is a powerful training procedure that has already been successfully used in pollen analyses to reconstruct past climatic conditions[11,12]. It is an improvement of the Weighted Averaging method that used the residual correlations in the species data sets, and is based on a multi-component analysis termed partial least squares regression[14,16]. We used the WA-PLS method as a linearity filter of the plant species-temperature relationship. A three-component WA-PLS model was selected on the basis of its low standard deviation of prediction error (SD = 1.02°C and 1.09°C for the training and validation [n = 5,136 independent surveys] data sets, respectively), low bias (mean of prediction error [*FrT* - *CrT*] = 0.02°C and -0.06°C for the training and validation data sets, respectively), high coefficient of determination between observed and predicted values (R^2 = 0.82 and 0.79 for the training and validation data sets, respectively), and the smallest number of 'useful' components[13]. We subsequently used the residuals $resM_1$ of this WA-PLS model to calibrate a new floristic model based on *floAss*: $resM_1 = g_{(floAss)}$ (model 2). We fitted this correlation with BRF regression techniques that incorporate interactions between plant species and the non-linear relationship between species and temperature. BRF is a non-parametric model that does not require the specification of a functional form, and provides good predictive accuracy without overfitting the data[15]. The BRF algorithm fits an average model from a collection of regression trees (5,000 in the present study). Each tree integrates a defined number of explanatory variables, which are randomly selected (fixed to 40 out of the 760 species, here). The BRF model explained 90% and 23% of the residual variation in the training and validation data sets, respectively. Finally, we combined the predictions of the WA-PLS and BRF models to infer the annual mean temperature provided from the floristic assemblage: *FrT* = model 1 - model 2. This original modeling approach significantly improved the relationship between mean annual temperatures

and floristic assemblages (R^2 reached 0.95 and 0.83 in the training and validation data sets, respectively) and reduced the error of predictions (in the training data set: median value = -0.31°C, $CI_{95\%}$ = [-1.06; 0.1], Wilcoxon paired signed-rank test: $P < 0.0001$; in the validation data set: median value = -0.1°C, $CI_{95\%}$ = [-0.66; 0.32], Wilcoxon paired signed-rank test: $P < 0.0001$) compared to the initial WA-PLS model.

All the computation were conducted on the *R* freeware[17] using both *pls*[18] and *randomForest*[19] *R* packages.

Method to infer the temperature buffering due to canopy cover.

Climate data used in the present study are based on interpolation methods[2,3] that infer climate conditions for each 1 km² (see descriptions of *CrT*, *T*, *P*, *TC* and *PC* variables in the Methods section) or 2,500 m² (see description of *THET* variable in the Methods section) spatial unit of the entire French territory. Although these interpolation methods account for the general cooler temperature observed in forest ecosystems than in open habitats for instance[2,3], such methods do not account for the local effect of forest canopy on sub-canopy temperature conditions[20,21]. Such local conditions are important to consider because sub-canopy temperatures near the ground define the climate really perceived by understory plants[22]. Considering the local climate or microclimate conditions when the impact of climate change on animal or plant species is investigated are increasingly underlined[23]. Canopy cover filters solar radiation penetrating inside the forest stand, and thus buffers (at least partly) the exposure of understory plant species to climate warming[3,24,25].

In the absence of direct measurements of sub-canopy temperatures for each of the 67,289 surveyed plant communities, we inferred sub-canopy temperature conditions from the microclim model[26]. This model is a mechanistic model predicting hourly climate conditions (e.g. above-ground profiles of air temperature, soil temperature, air humidity and soil humidity) from the local environmental (notably shading by vegetation) and monthly macroclimate conditions. The microclim

model has been validated for Australia[27], and has been recently used to predict hourly baseline climate conditions throughout the world[26]. Here, we did not use these global predictions that we considered too coarse to be representative of the sub-canopy temperature conditions during the 1987-2008 period that we investigated. Instead, we ran the microclim model using forest stand characteristics (canopy cover and height) and climate conditions (estimated from spatio-temporal climate model[2-5]) observed and predicted, respectively, at the year and location of a subset of 45,806 floristic surveys (all from the IGN-IFN database[8]) for which all the data necessary to compute sub-canopy temperature conditions was available (Supplementary Table 5). Hourly in-situ temperature predictions near the ground (i.e. at 0.1 m height) considering and not considering the observed canopy cover were computed for each month of the year of each floristic observation, and then averaged to achieve mean annual temperatures. We finally computed the temperature buffering effect due to canopy cover as the difference between the mean annual temperatures near the ground considering and not considering the canopy cover (Supplementary Fig. 11).

Supplementary References

1. Hutcheon, J. A., Chiolero, A. & Hanley, J. A. Random measurement error and regression dilution bias. *BMJ* **340**, c2289 (2010).

2. Bertrand, R. *et al.* Changes in plant community composition lag behind climate warming in lowland forests. *Nature* **479**, 517-520 (2011).

3. Bertrand, R. *Spatio-temporal response of the forest vegetation to climate warming - Assessment of the vegetation reshuffling and characterisation of the effect of ecological and geographical factors modulating this process at the species and community scales*, Ph.D. thesis, AgroParisTech (2012).

4. Harris, I., Jones, P. D., Osborn T. J. & Lister, D. H. Updated high-resolution grids of monthly climatic observations–the CRU TS3.10 Dataset. *Int. J. Climatol.* **34**, 623-642 (2014).

5. New, M., Lister, D., Hulme, M. & Makin, I. A high-resolution data set of surface climate over global land areas. *Clim. Res.* **21**, 1-25 (2002).

6. Piedallu, C., Gégout, J. C., Bruand, A. & Seynave, I. Mapping soil water holding capacity over large areas to predict potential production of forest stands. *Geoderma* **160**, 355-366 (2011).

7. Hengl, T. *et al.* SoilGrids1km - global soil information based on automated mapping. *PLoS One* **9**, e105992 (2014).

8. Robert, N. *et al.* in *National Forest Inventories: pathways for common reporting* (eds Tomppo, E.; Gschwantner, T.; Lawrence, M. & McRoberts, R. E.) 207-221 (Springer Netherlands, 2010).

9. Dormann, C. F. *et al.* Methods to account for spatial autocorrelation in the analysis of species distributional data: a review. *Ecography* **30**, 609-628 (2007).

10. Kühn, I. Incorporating spatial autocorrelation may invert observed patterns. *Divers. Distrib.* **13**, 66-69 (2007).

11. Heikkila, M. & Seppa, H. A 11,000 yr palaeotemperature reconstruction from the southern boreal zone in Finland. *Quat. Sci. Rev.* **22**, 541-554 (2003).

12. Herzschuh, U., Birks, H. J. B., Mischke, S., Zhang, C. J. & Bohner, J. A modern pollen-climate

calibration set based on lake sediments from the Tibetan Plateau and its application to a Late Quaternary pollen record from the Qilian Mountains. *J. Biogeogr.* **37**, 752-766 (2010).

13. Birks, H. J. B. Numerical tools in palaeolimnology – progress, potentialities, and progress. *J. Palaeolimnol.* **20** (1998).

14. ter Braak, C. J. F. & Juggins, S. Weighted averaging partial least squares regression (WA-PLS): an improved method for reconstructing environmental variables from species assemblages. *Hydrobiologia* **269**, 485-502 (1993).

15. Breiman, L. Random forests. *Mach. Learn.* **45**, 5-32 (2001).

16. ter Braak, C. J. F. & van Dam, H. Inferring pH from diatoms: a comparison of old and new calibration methods. *Hydrobiologia* **178**, 209-223 (1989).

17. R Core Team. *R: A Language and Environment for Statistical Computing* (R Foundation for Statistical Computing, Vienna, Austria) at <http://www.R-project.org/> (2011).

18. Mevik, B. H. & Wehrens, R. pls: Partial Least Squares Regression (PLSR) and Principal Component Regression (PCR). *J. Stat. Soft.* **18**, 1-24 (2007).

19. Liaw, A. & Wiener, M. Classification and Regression by randomForest. *R news* **2**, 18-22 (2002).

20. Spurr, S. H. Local Climate in the Harvard Forest. *Ecology* **38**, 37-46 (1957).

21. Graae, B. J. *et al.* On the use of weather data in ecological studies along altitudinal and latitudinal gradients. *Oikos* **121**, 3-19 (2012).

22. Jones, H. G. *Plants and microclimate: a quantitative approach to environmental plant physiology*, 3rd edn (Cambridge university press, 2013).

23. Potter, K. A., Arthur Woods, H. & Pincebourde, S. Microclimatic challenges in global change biology. *Glob. Change Biol.* **19**, 2932-2939 (2013).

24. De Frenne, P. *et al.* Microclimate moderates plant responses to macroclimate warming. *Proc. Natl. Acad. Sci. U.S.A.* **110**, 18561-18565 (2013).

25. Stevens, J. T., Safford, H. D., Harrison, S. & Latimer, A. M. Forest disturbance accelerates

thermophilization of understory plant communities. *J. Ecol.* (2015).

26. Kearney, M. R., Isaac, A. P. & Porter, W. P. *microclim*: Global estimates of hourly microclimate based on long-term monthly climate averages. *Scientific data* **1**, 1-9 (2013).

27. Kearney, M. R. *et al.* Microclimate modelling at macro scales: a test of a general microclimate model integrated with gridded continental scale soil and weather data. *Methods Ecol. Evol.* **5**, 273-286 (2014).
